# Transcriptomic analysis of paired healthy human skeletal muscles to identify modulators of disease severity in DMD

**DOI:** 10.3389/fgene.2023.1216066

**Published:** 2023-07-27

**Authors:** Shirley Nieves-Rodriguez, Florian Barthélémy, Jeremy D. Woods, Emilie D. Douine, Richard T. Wang, Deirdre D. Scripture-Adams, Kevin N. Chesmore, Francesca Galasso, M. Carrie Miceli, Stanley F. Nelson

**Affiliations:** ^1^ Department of Human Genetics, David Geffen School of Medicine, University of California, Los Angeles, Los Angeles, CA, United States; ^2^ Center for Duchenne Muscular Dystrophy at UCLA, Los Angeles, CA, United States; ^3^ Department of Microbiology, David Geffen School of Medicine and College of Letters and Sciences, University of California, Los Angeles, Los Angeles, CA, United States; ^4^ Department of Pediatrics, David Geffen School of Medicine, University of California, Los Angeles, Los Angeles, CA, United States; ^5^ Department of Neurology, David Geffen School of Medicine, University of California, Los Angeles, Los Angeles, CA, United States; ^6^ Department of Pathology and Laboratory Medicine, David Geffen School of Medicine, University of California, Los Angeles, Los Angeles, CA, United States

**Keywords:** muscle, transcriptomics, DMD, muscle susceptibility, gene expression, single nuclei RNAseq

## Abstract

Muscle damage and fibro-fatty replacement of skeletal muscles is a main pathologic feature of Duchenne muscular dystrophy (DMD) with more proximal muscles affected earlier and more distal affected later in the disease course, suggesting that different skeletal muscle groups possess distinctive characteristics that influence their susceptibility to disease. To explore transcriptomic factors driving differential gene expression and modulating DMD skeletal muscle severity, we characterized the transcriptome of vastus lateralis (VL), a more proximal and susceptible muscle, relative to tibialis anterior (TA), a more distal and protected muscle, in 15 healthy individuals using bulk RNA sequencing to identify gene expression differences that may mediate their relative susceptibility to damage with loss of dystrophin. Matching single nuclei RNA sequencing data was generated for 3 of the healthy individuals, to infer cell composition in the bulk RNA sequencing dataset and to improve mapping of differentially expressed genes to their cell source of expression. A total of 3,410 differentially expressed genes were identified and mapped to cell type using single nuclei RNA sequencing of muscle, including long non-coding RNAs and protein coding genes. There was an enrichment of genes involved in calcium release from the sarcoplasmic reticulum, particularly in the myofibers and these myofiber genes were higher in the VL. There was an enrichment of genes in “Collagen-Containing Extracellular Matrix” expressed by fibroblasts, endothelial, smooth muscle and pericytes, with most genes higher in the TA, as well as genes in “Regulation Of Apoptotic Process” expressed across all cell types. Previously reported genetic modifiers were also enriched within the differentially expressed genes. We also identify 6 genes with differential isoform usage between the VL and TA. Lastly, we integrate our findings with DMD RNA sequencing data from the TA, and identify “Collagen-Containing Extracellular Matrix” and “Negative Regulation Of Apoptotic Process” as differentially expressed between DMD compared to healthy. Collectively, these findings propose novel candidate mechanisms that may mediate differential muscle susceptibility in muscular dystrophies and provide new insight into potential therapeutic targets.

## 1 Introduction

Duchenne muscular dystrophy (DMD) is the most common progressive muscular dystrophy with childhood onset, and is caused by loss of function mutations in *DMD* (Hoffman et al., 1987), leading to profound weakness and premature death, mainly from cardiorespiratory failure. *DMD* encodes dystrophin, which plays a critical structural role in skeletal and cardiac muscle fibers by linking the intra-myofiber F-actin of the Z-disk to the extracellular matrix through binding components of the dystrophin-associated glycoprotein complex at the muscle membrane (Hoffman et al., 1987; Way et al., 1992). Absence of dystrophin in skeletal muscle leads to greater susceptibility to damage from contraction-induced injury (Petrof et al., 1993), resulting in leakage of calcium into the myofiber with a plethora of downstream consequences ultimately leading to myofiber death and replacement with fat and fibrosis. Fibroblasts, immune cells, and muscle stem cells are expanded, changing the extracellular matrix (Scripture-Adams et al., 2022). A large number of other muscular dystrophies have had their genetic basis decoded and many are components of the dystrophin-glycoprotein complex (Cohen et al., 2021), including Limb-girdle muscular dystrophies (LGMDs) with similar patterns of muscle loss from proximal to more distal.

While DMD is always degenerative and leads to premature death, variation in disease progression between individuals in DMD has been used to identify genetic factors correlated with disease severity or progression. Disease severity is mitigated with residual dystrophin expression which usually results in slowing of disease progression (Fanin et al., 1995). However, even in cases of siblings with DMD who have the same *DMD* mutation, there can be discordance in the progression (Pettygrove et al., 2014), indicating that environmental or other genetic factors may modify disease severity. Various studies use variability in age at loss of ambulation (LOA) (Pegoraro et al., 2011; Flanigan et al., 2013; Bello et al., 2016; Weiss et al., 2018; Spitali et al., 2020) to identify variants associated with disease progression.

The overall pattern of sequentially affected muscles in DMD is highly similar across affected individuals and describes a distinctive pattern of progression with more proximal muscles affected earlier than more distal muscles (Rooney et al., 2020), suggesting that constitutive differences in the formation of those muscle groups encode factors that influence relative myofiber susceptibility to damage. Therefore, the study of healthy muscle may provide insights into susceptibility mechanisms in disease. An extreme example of protected striated muscles in DMD across multiple species are the extraocular muscles (EOM) (Karpati et al., 1988; Valentine et al., 1990; Kaminski et al., 1992). However, the functional requirements of EOM are substantially different from limb skeletal muscle. EOM have multiple innervated fibers, compartmentalization of layers with different fiber types, expression of EOM-specific myosin isoforms (encoded by *MYH13*, *MYH15*), and partial retention of embryonic and neonatal myosin expression in mature fibers (Porter, 2002). Differential gene expression studies in mouse (Porter et al., 2001) and rat (Fischer et al., 2002) highlighted calcium homeostasis, mitochondrial genes, lipid catabolism, immune processes, apoptosis, and extracellular matrix.

Here we study healthy muscle tissue from vastus lateralis (VL) and tibialis anterior (TA), to identify genes that alter myofiber susceptibility to fibrofatty replacement in DMD individuals, using paired samples from 15 donors to control for interindividual and age differences. While TA has a much more modest degree of protection from disease progression than EOM, TA is substantially and consistently protected from ongoing muscle damage in DMD relative to VL from longitudinal imaging and spectroscopy data of children with DMD (Rooney et al., 2020). We reasoned that the differential expression analysis of VL and TA in healthy individuals would provide insight into protective mechanisms relevant in the absence of dystrophin. The difference in progression is substantial. VL progresses faster than TA with an about 8.5-year longer time for the TA to attain similar levels of damage as the VL (Rooney et al., 2020). In this transcriptomic study, we sampled VL and TA at a single timepoint from healthy young adult volunteers. We report differentially expressed genes and map differentially expressed genes to specific intra-muscular cell types using single nuclei analyses.

## 2 Materials and methods

### 2.1 Muscle biopsies

Fifteen healthy individuals (age range 18–26 years) with no history of muscle or other chronic or acute disease were consented on UCLA protocol IRB#18-001366. Eight ambulatory DMD patients with a confirmed nonsense *DMD* mutation were consented on UCLA protocol IRB#11-001087 (age range 2–7 years). All biopsies were obtained using a Vacora (Bard) vacuum-assisted core needle from the VL and TA as previously described (Barthelemy et al., 2020). In brief, before the biopsies, the participant’s leg was observed via ultrasound to ensure that the muscle showed no excess fat or blood vessels nearby. VL sample was obtained from about two-thirds of the muscle length, and the TA from about one-third of the muscle length. We chose muscle pieces that had similar muscle appearance without visible connective tissue to reduce sample variability. Each needle muscle biopsy core (about 125 mg) was dissected into about 25 mg pieces and flash frozen in liquid nitrogen within tissue cassettes within 5 min of excision and stored in liquid nitrogen until RNA extraction or sectioning for histological examination.

### 2.2 RNA sequencing

Frozen skeletal muscle (8–25 mg) was homogenized on ice in 500 µL of Trizol for RNA extraction using standard protocols (Lee et al., 2020). RNA quality was recorded by the RNA integrity number (RIN) using the Agilent RNA 6000 Nano chips. Healthy muscle RNA samples with RIN above 7 and DMD muscle RNA samples with RIN above 4 were used to prepare cDNA libraries with ribosomal RNA depletion using the KAPA RiboErase Kit (HMR) (Roche). About 50 million 150-151 bp paired-end RNA sequencing (RNAseq) reads were generated per RNA sample using Illumina Novaseq 6000 S4. Sequencing reads were aligned to GRCh38 (Ensembl 105, Gencode v39) using STAR 2.6.0c (Dobin et al., 2012; Lee et al., 2020). Data quality control included alignment metrics (ribosomal and globin RNA, aligned and unmapped reads, sequencing depth), hierarchical clustering, principal component analysis and Pearson correlation.

### 2.3 Single nuclei isolation and RNA sequencing

Single nuclei were isolated from a subset of 3 paired male healthy VL and TA frozen muscle and sequenced using the 10X Genomics platform as described previously (Scripture-Adams et al., 2022). Six to twelve 40 µM cross sections of frozen muscle biopsies were collected in a sterile tube to estimate a total of 3 mg of skeletal muscle, dounced with two cycles of strokes (one with a loose douncer followed by one with a tight douncer) in 1% bovine serum albumin (BSA) in phosphate-buffered saline (PBS) with 100 U/mL of type IV collagenase and 0.5 U/µL RNAse inhibitor, and stained with 10 μg/mL DAPI. The nuclei were sorted by fluorescence-activated cell sorting (FACS) to separate from debris and create a pure nuclear preparation prior to library preparation. 10X Chromium Single cell 3′ v3 libraries were prepared and sequenced on Illumina Novaseq 6000 S2 (2 × 50 bp) (10X Genomics). Single nuclei RNA sequencing (snRNAseq) reads were aligned to GRCh38 (Ensembl 105, Gencode v39) using Cell Ranger (10X Genomics). Data was aggregated for downstream processing and analysis. Initial cell clustering was performed using k-means within Cell Ranger (10X Genomics). Nuclear doublets were identified using DoubletFinder (version 2.0.3) (McGinnis et al., 2019) with a doublet rate of detection of 15%. Doublets as well as nuclei with 200 or fewer unique molecular identifiers (UMI), were excluded from downstream analysis. Re-clustering was performed after data filtering, and clustered nuclei populations were identified using known cell-type marker genes via Loupe Browser (version 6.0.0) (10X Genomics). Downstream analysis and statistical testing of differentially expressed genes across cell types was performed using the R package Seurat (version 4.0.2) (Hao et al., 2021) and the Wilcoxon statistical test. UMI-normalized average expression across cell types was obtained from Seurat’s AverageExpression function, which returns the average number of transcripts per 10,000 transcripts (TP10K).

### 2.4 Cell deconvolution using single nuclei RNA sequencing

Raw bulk RNAseq read counts for were obtained from the STAR alignment (version 2.6.0c) (Dobin et al., 2012) and batch-corrected for the two sequencing runs using CombatSeq (sva version 3.38.0) (Zhang et al., 2020). Differential gene expression analysis across cell types in the snRNAseq dataset identified statistically significant (adjusted *p*-value <0.05) marker genes for each cell type. Highly specific markers for a specific cell type were defined as those expressed in less than 10% (for large cell populations) or 1% (for small cell populations) of the other cell types. A list of 69 cell-specific genes was obtained after further manual curation. Estimated cell proportions for each sample were obtained with CIBERSORTx (Newman et al., 2019) using the average expression of these 69 cell-specific genes. The parameters used for CIBERSORTx were: Job type = Impute Cell Fractions, Batch correction = disabled, Disable quantile normalization = true, Run mode = relative, Permutations = 100.

### 2.5 Differential gene expression analysis

The R package DESeq2 (version 1.30.1) (Love et al., 2014) was used to perform differential gene expression analysis using the raw read counts. The covariates included in the healthy VL *versus* TA analysis design were: participant study ID, RIN, and batch. The covariates included in the DMD *versus* Healthy analysis design were: batch, RIN, age, and sex. Multiple testing adjustment was done within DESeq2 using Benjamini–Hochberg for a false discovery rate (FDR) of less than 0.05.

Functional enrichment analysis of differentially expressed genes was performed for all differentially expressed genes (independent of their direction of highest expression) using EnrichR (https://maayanlab.cloud/Enrichr/, (Chen et al., 2013)), with all expressed genes included in the DESeq2 analysis as background. For the genes differentially expressed between VL and TA, we tested 4,701 terms from GO Biological Process 2023, and 408 terms from GO Cellular Component 2023. For genes differentially expressed between DMD and healthy, we tested 3,133 terms from GO Biological Process 2023, and 272 terms from GO Cellular Component 2023. Significant gene ontology (GO) terms (adjusted *p*-value <0.05) for the VL *versus* TA analysis were further summarized with ReviGO (http://revigo.irb.hr/, (Supek et al., 2011)) with the following parameters: dispensability threshold = 0.5, GO metric = adjusted *p*-value (lower value is better), remove obsolete GO terms = yes, species = Whole UniProt database, similarity measure = SimRel.

ENCODE_and_ChEA_Consensus_TFs_from_ChIP-X enrichment category within EnrichR (Chen et al., 2013) was used to identify transcription factors (104 transcription factors tested) that putatively bind to the differentially expressed genes. Pathway enrichment of druggable genes higher in the VL was performed using EnrichR (Chen et al., 2013) KEGG 2021 Human enrichment category (250 terms tested).

### 2.6 Differential isoform usage analysis

The VL and TA raw data was aligned using the Kallisto app (Kallisto Quantification version 2.0.2, Kallisto 04.46.1) on the DNAnexus platform pipeline (Bray et al., 2016) to obtain counts and relative abundance (TPM) for each transcript (Gencode v39, Ensembl 105).

Differential isoform usage analysis between VL and TA was performed using the IsoformSwitchAnalyzeR (version 1.12.0) R package (Vitting-Seerup and Sandelin, 2017). The design matrix included: sex, sample RIN, and batch. Gencode v39 primary assembly annotation and transcripts were used to generate the switch list. Isoforms were prefiltered before testing for differential isoform usage using the following parameters: gene expression cutoff = 0.1 and isoform expression cutoff = 0, and genes with only one isoform were excluded. Isoform switch testing was done using DEXSeq (version 1.36.0) (Anders et al., 2012) within the IsoformSwitchAnalyzeR package, and correction for confounding factors indicated in the design matrix was performed simultaneously via the limma package (version 3.46.0) (Ritchie et al., 2015). The isoform switch analysis was limited to: switching genes (genes with at least one isoform significantly differentially used), genes with consequence potential (with isoforms differentially used in opposing directions, i.e., one with increased and one with decreased usage), and isoforms with at least two isoforms significantly differentially used (alpha<0.05), and a difference in isoform usage between muscles of at least 0.01 (1%).

### 2.7 Immunofluorescence

Skeletal muscle tissue was cross-sectioned at 10 µM thickness after equilibration at −22°C in a cryostat and then stored at −80°C until immunofluorescence was performed. Slides were acclimated to room temperature and sections were circled using a hydrophobic barrier pen. For actinin-3 staining, sections were fixed with PFA 4% for 10 min and permeabilized using 0.5% Triton-X for 10 min at room temperature. Sections were treated with TrueBlack Lipofuscin Autofluorescence Quencher before blocking with 3% BSA/10% goat serum in PBS for 1 h at room temperature, and then incubated in primary antibody in blocking solution overnight at 4°C in a humidified chamber. For nebulin staining, unfixed samples were permeabilized with 0.5% Triton-X for 10 min at room temperature. Samples were blocked with 3% BSA for 1 h at room temperature and incubated in primary antibody solution in 3% BSA overnight at 4°C in a humidified chamber. Primary antibodies used were: monoclonal anti-actinin-3 (Abcam, ab68204, 1.61 μg/mL), monoclonal anti-myosin skeletal slow (Sigma, m8421, 4.8 μg/mL), mouse anti-NEB143(3F4) ((Lam et al., 2018), 149 μg/mL), rabbit anti-MYH1 (Sigma, SAB2104768, 5–10 μg/mL), rat anti-MYH2 clone 8F72C8 (EMD Millipore, MABT848, 40 μg/mL). Sections were then incubated in secondary antibody in PBS for 2 h at room temperature. For nebulin staining, secondary antibody for rat and mouse were cross adsorbed to prevent cross-reactivity: Goat anti-Mouse IgG (H + L) Cross-Adsorbed Secondary Antibody, DyLight 550 (Invitrogen, SA5-10173, 1:500), and Cy5 AffiniPure Donkey Anti-Rat IgG (H + L) (Jackson Laboratories, 712-175-153, 1:300). Slides were mounted in Antifade Mounting Medium with DAPI (Vectashield, H-1200-10). Images were obtained using a fluorescent microscope and processed using ImageJ (Schneider et al., 2012) (release 1.53c).

## 3 Results

### 3.1 Identification of transcriptional differences between VL and TA

**FIGURE 1:**
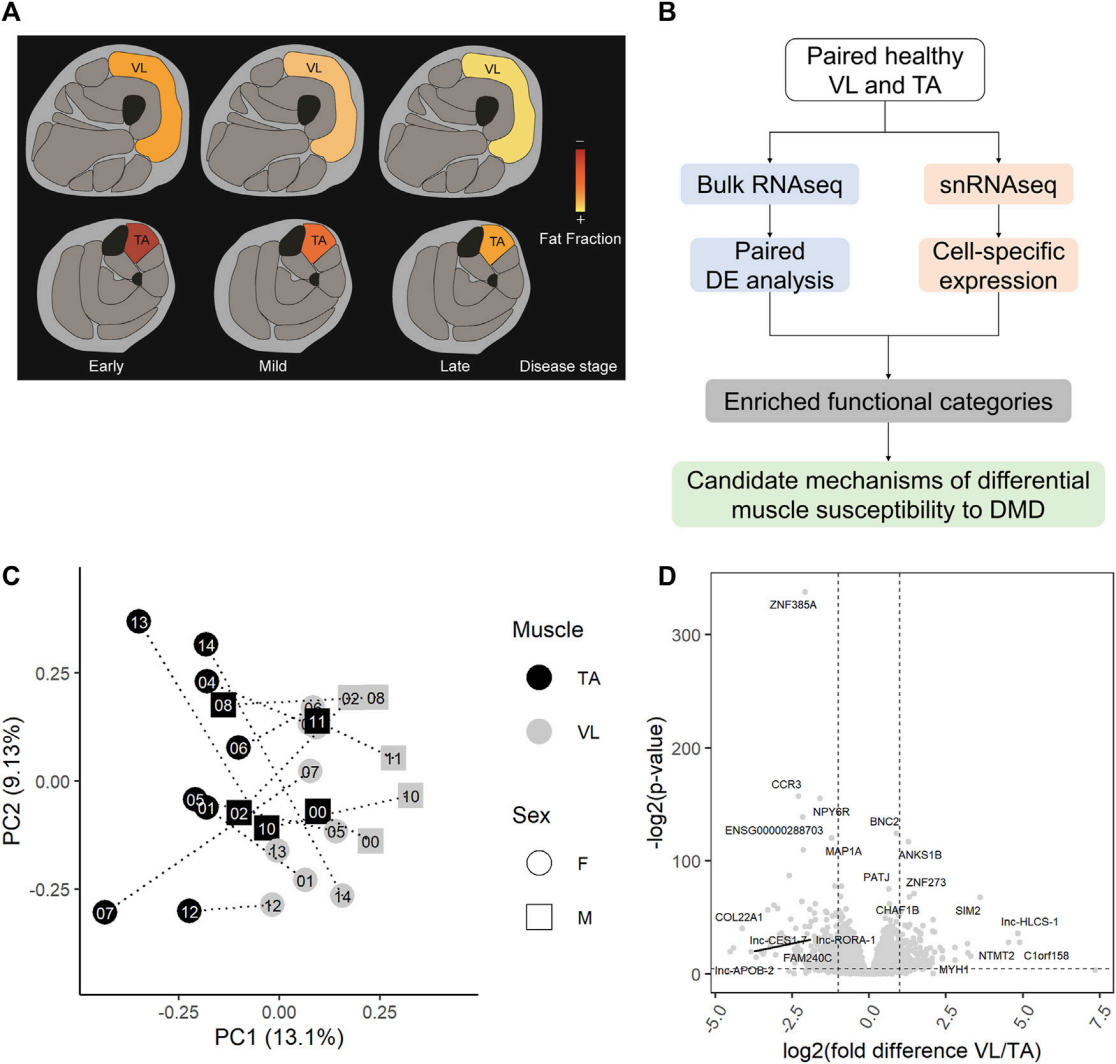
Healthy VL and TA transcriptomes are highly different. **(A)** Representation of the differential progression of vastus lateralis (VL) and tibialis anterior (TA) in DMD. The color scale indicates the progression from early stages of DMD where muscle fat fraction is minimal (illustrated in red), to late stages where muscle fibers are completely replaced by fat (illustrated in yellow). **(B)** Workflow for the identification of candidate mechanisms mediating differential muscle susceptibility to DMD. **(C)** The first two principal components (PC1 and PC2) are shown for batch-corrected normalized RNAseq data expression of all expressed genes (*n* = 22,414) across the 27 muscle samples. **(D)** Volcano plot for all 22,414 genes tested for differential expression. Dashed lines depict a fold change of 2 and a *p*-value of 0.05. For each muscle, the top 5 differentially expressed genes by fold difference and the top 5 genes by *p*-value are labeled.

Because of the substantial difference in the rate of progression/damage of VL and TA in DMD (Figure 1A), we characterized the intrinsic transcriptomic profiles of paired healthy VL and TA using RNAseq and snRNAseq to reveal candidate mechanisms that may underlie this differential susceptibility to DMD (Figure 1B). VL and TA biopsies were sampled from each of 15 healthy young adults during the same procedure. Extraction of RNA from frozen skeletal muscle was adequate with an average RIN of 8 across all samples (range 7.1–8.7), and an average of 54 million sequencing reads were obtained per sample (range 45–76 million reads). One sample that had lower sequencing depth, and two samples that were outliers by hierarchical clustering and had relatively lower correlation with the overall dataset were excluded. A total of 27 healthy muscle samples (26 paired VL-TA, 1 unpaired VL) were used for further analysis. Two-dimensional principal component analysis (PCA) on the expression of all 22,414 expressed genes among 27 samples demonstrates that RNAseq data cluster predominantly by muscle type and that muscles from the same individual do not cluster together (Figure 1C). This indicates that there is more expression similarity between unrelated individuals in either the VL or TA than within an individual, or alternatively stated there are more intraindividual gene expression differences between VL and TA than interindividual differences from genetic variation.

Using DESeq2 (Love et al., 2014) with a paired analysis design, we identified a large set of 3,410 significantly differentially expressed genes (Supplementary Table S1), or 15.2% of all genes, demonstrating a substantial number of gene expression differences between the skeletal muscle groups. When we randomize participant IDs within muscle groups such that samples are no longer paired, and test for differential gene expression, we do not observe as many differentially expressed genes as we do with a paired analysis (empirical *p*-value = 0, *n* = 1,000 permutations). That is, our paired analysis of both muscles from the same individuals allowed us to identify a larger number of differentially expressed genes than we would have with an unpaired design. The most statistically significant differentially expressed gene was the transcription factor *ZNF385A*, and other top differentially expressed genes (by fold difference or *p*-value) included *MYH1*, *COL22A1*, the transcription factors *BNC2*, *SIM2* and *ZNF273*, and the non-coding RNAs lnc-HLCS-1, lnc-CES1-7, lnc-APOB-2, and lnc-RORA-1 (Figure 1D). Genes classified as protein coding by the Ensembl automatic annotation system were more likely to be differentially expressed, comprising 82% of all differentially expressed genes, but a substantial number of long noncoding RNAs (lncRNAs) are differentially expressed between the muscles (Supplementary Table S1).

Gene ontology analysis on all differentially expressed genes revealed an enrichment of 619 biological processes and 85 cellular component categories (Supplementary Table S2A). After summarization of redundant terms with ReviGO, the summarized GO term list is comprised of 75 biological processes and 28 cellular component GO terms (Supplementary Table S2B). We further focused on GO terms with over 10 genes, such that we could examine a larger number of genes contributing to the enrichment, resulting in a list of 58 biological processes and 21 cellular components. For each GO category, we ranked them by adjusted *p*-value, and then selected 6 relevant categories (Table 1) based on their involvement in muscle function and the dystrophic pathology.

**TABLE 1:** Differentially expressed genes are enriched within regulation of calcium, extracellular matrix and regulation of apoptosis. Significant gene ontology (GO) terms enriched among all 3,410 differentially expressed genes (independent of the muscle where they are highest expressed) are shown. EnrichR significant GO terms for biological process (BP) and cellular component (CC) were summarized using ReviGO. Selected most relevant and significant terms with over 10 genes are shown. The top 15 genes by average fold difference between VL and TA are shown.

GO	GO term	Count	Adjusted *p*-value	Odds ratio	Top 15 genes by average fold difference
BP	Cytoplasmic Translation	71	9.77E-36	18.28	*RPS15A, RPL21, RPL35, RPL9, RPL39, RPL6, RPL29, RPS3A, RPL7, RPL27, RPLP0, MRPS12, RPS18, RPS13, RPS15*
	Muscle Contraction	48	7.56E-15	6.92	*MYH1, RYR2, MYL6B, MYH11, MYH6, TPM1, MYH2, MYLK, MYLK2, MYH3, MYH4, TPM4, OXTR, MYL1, TPM3*
	Regulation Of Cell Migration	121	2.46E-14	2.75	*TBX5, EPPK1, TNC, FGF9, CCR1, NKD1, PLXNA4, SERPINE1, NTRK3, SH3RF2, STC1, SFRP1, TPM1, TWIST2, PAK1*
	Regulation Of Cell Adhesion	45	3.97E-07	3.41	*TNC, DACT2, PLXNA4, TPM1, PLXNB1, ADAM22, DLL1, SRC, PDE3B, MYADM, EPHA4, EPHA2, TGFBI, PPP3CA, TGM2*
	Regulation Of Apoptotic Process	139	4.96E-06	1.76	*EGR3, COMP, ACTN3, EGR1, SH3RF2, ANGPTL4, FRZB, GATA6, SFRP1, MLLT11, ACTN1, TENT5B, GADD45G, MPO, SMAD6*
	Regulation Of Release Of Sequestered Calcium Ion Into Cytosol By Sarcoplasmic Reticulum	13	9.98E-05	9.05	*RYR2, CASQ1, CASQ2, SLC8A1, GSTO1, ANK2, CACNA1C, DMD, CALM2, CALM1, TRDN, PDE4D, ATP1A2*
CC	Focal Adhesion	156	7.17E-33	4.1	*CNN1, ACTN3, TNC, CSRP1, ACTN1, SLC9A1, SPRY4, FLNA, MCAM, BCAR3, ITGA5, THY1, LAYN, CD9, SRC*
	Large Ribosomal Subunit	41	3.52E-24	28.79	*RPL21, RPL35, RPL9, RPL39, RPL6, RPL29, RPL7, RPL27, RPLP0, RPL38, RPL37A, RPL7A, RPL4, RPL14, RPL24*
	Actin Cytoskeleton	105	8.76E-17	3.18	*CNN1, ACTN3, SORBS2, TPM1, ACTN1, PAK1, FLNA, MYLK, ABLIM1, CD274, MYL2, CNN2, TPM4, MYADM, MYLK3*
	Collagen-Containing Extracellular Matrix	88	2.10E-09	2.43	*ACAN, COMP, TNC, LEFTY2, COL28A1, COL21A1, CCN2, SERPINE1, COLQ, ANGPTL4, INHBE, SLPI, SFRP1, SERPINB8, NCAM1*
	Sarcoplasmic Reticulum	25	3.30E-09	7.76	*RYR2, ATP2A1, SLN, THBS1, ATP2A2, DMPK, CASQ1, ATP2A3, STRIT1, JPH1, CASQ2, JSRP1, ITPR1, S100A1, ITPR3*
	Sarcolemma	21	6.01E-05	4.04	*RYR2, ATP1A1, CAV2, SLC8A1, CACNG1, ANK2, SLC2A5, DMD, CAV3, SGCB, SYNC, CAV1, POPDC3, RYR1, DYSF*

Italic values are the gene names (gene symbols).

### 3.2 Cell type differences in VL and TA

Fiber type composition varies between skeletal muscles in mice (Terry et al., 2018) and humans (Abbassi-Daloii et al., 2023). In humans, VL has a larger portion of fast myofibers than TA (Edgerton et al., 1975; Jakobsson et al., 1991), whereas in mouse, the TA is composed entirely of fast myofibers (Hämäläinen and Pette, 1993; Scripture-Adams et al., 2022). In DMD, the differential disease susceptibility between different skeletal muscles has been partly attributed to the higher proportion of fast fibers, which are more susceptible to damage in the disease course than slow fibers (Webster et al., 1988). These differences in cell composition may contribute to the differential disease susceptibility and be reflected in the observed transcriptomic differences. To assess the contribution of cell composition to gene expression, we performed snRNAseq of nuclei dissociated from a small subset of the healthy individuals. After excluding doublets and nuclei with less than 200 UMI, the VL and TA dataset consists of 14,887 single nuclei (5,151 VL and 9,736 TA) with a median of 386 genes and 568 UMI per nucleus, and with a total of 25,248 genes detected within all of the nuclei.

**FIGURE 2:**
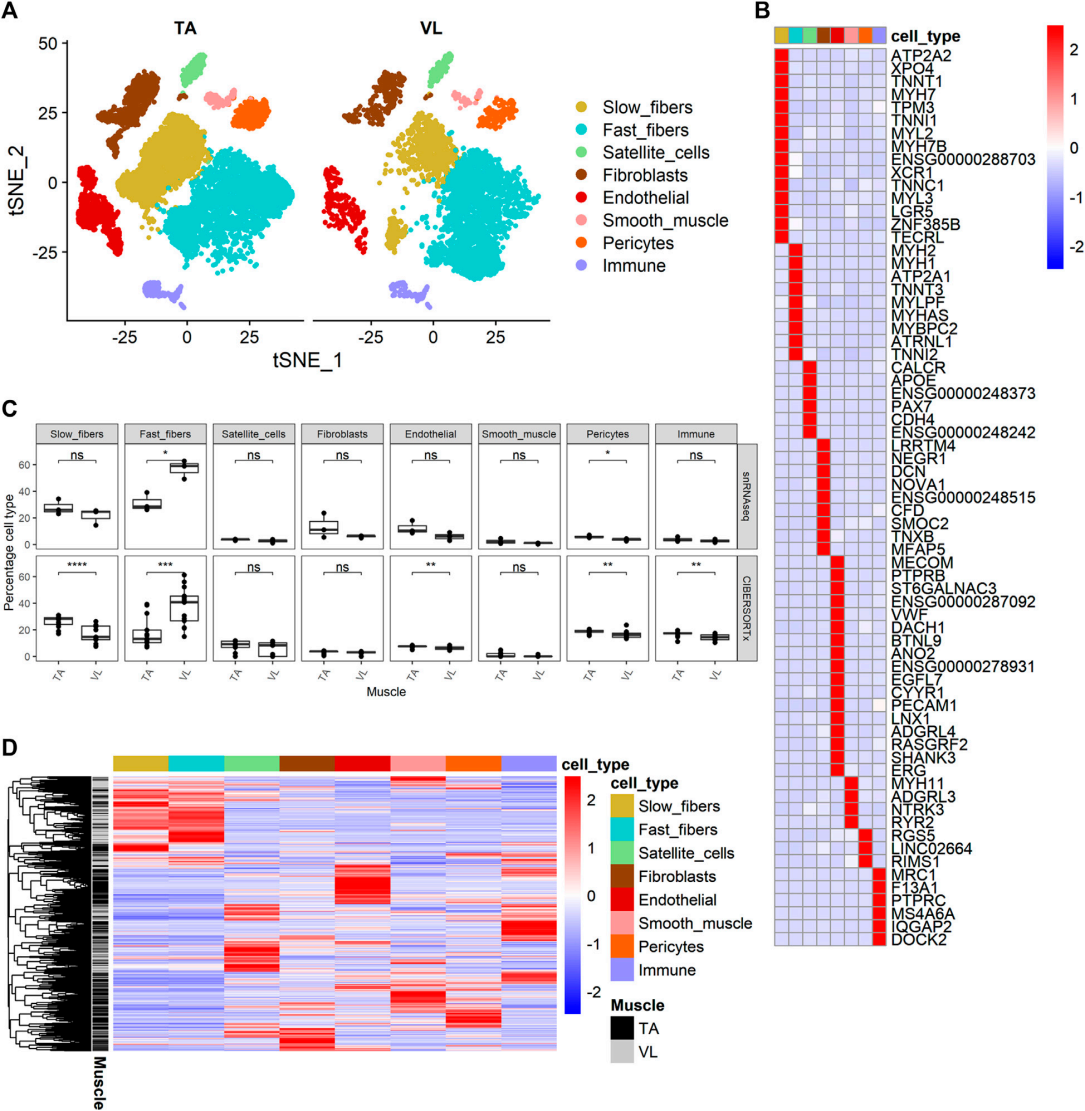
Identification of cell composition differences and cell source of differential gene expression. **(A)** t-SNE projection of 14,887 nuclear transcriptomes from 3 TA and 3 VL paired healthy muscles. **(B)** Heatmap of expression of 69 cell-specific marker genes of the major cell types identified. **(C)** Percentage of cell types from snRNAseq (top) and percentage of cell types inferred by CIBERSORTx (bottom) for paired VL and TA samples using the 69 specific markers in **(B)**. Paired *t*-test, *: p ≤ 0.05, **: p≤0.01, ***: *p* < 0.001,****: *p* < 0.0001. **(D)** Heatmap of expression of 3,221 genes differentially expressed between VL and TA in the main cell types identified in (A).

Clustering analysis resulted in the identification of 8 known major cell types (Figure 2A) with distinct transcriptomes (refer to Supplementary Table S2C for a list of positive marker genes). We compared the proportion of each major cell type within VL and TA. Consistent with previous reports, the three VL samples had a higher proportion of fast fibers compared to TA (paired *t*-test *p* = 4.25E-02, average fold difference 1.82) (Figure 2C). The 3 TA samples had 1.29 times as many slow fibers, although this difference was not statistically significant in our snRNAseq dataset (paired *t*-test *p* = 2.52E-01) (Figure 2C). Performing snRNAseq on a subset of samples allows us to infer cell composition in bulk RNAseq. By integrating bulk RNAseq and snRNAseq, we can explore the transcriptomic profile of our large dataset taking into consideration if the gene is specifically expressed in just 1 cell type, and thus map the differential expression of some genes to cell type. The snRNAseq dataset was also used to infer the percentage of all major cell types across our larger bulk RNAseq dataset. For this, we used CIBERSORTx (Newman et al., 2019) to deconvolute the bulk RNAseq data with 69 marker genes that we identified and define as being uniquely expressed within only one of the 8 major cell types (Figure 2B). Overall, the percentage of cell types inferred by CIBERSORTx agreed with those observed by snRNAseq in the six samples with both data types (Pearson correlation = 0.81), and we can infer from bulk RNAseq that TA has a larger portion of slow fibers than VL (paired *t*-test *p* = 3.69E-05, average fold difference 1.54) and VL has a higher portion of fast fibers (paired *t*-test *p* = 1.93E-04, average fold difference 2.07) (Figure 2C). We also observed a slight but significant increase in the percentage of endothelial (paired *t*-test *p* = 4.40E-03, average fold difference 1.16), pericytes (paired *t*-test *p* = 7.22E-03, average fold difference 1.14), and immune (paired *t*-test *p* = 3.63E-03, average fold difference 1.16) cells in TA compared to VL. The higher percentage of endothelial cells and pericytes in the TA is suggestive of a higher capillarity density compared to the VL. Similar differences in capillarity density across leg muscles have been reported previously, with a higher density in the lower leg gastrocnemius lateralis compared to the upper leg semitendinosus muscle (Abbassi-Daloii et al., 2023).

### 3.3 Mapping of differentially expressed genes to specific cell types within skeletal muscles

We next sought to identify the cellular source of differentially expressed genes identified by bulk RNAseq by interrogating their relative expression across the 8 major cell types identified in healthy human muscle. Out of 3,410 differentially expressed genes observed within the bulk RNAseq data, 3,221 (94.4%) were also observed in our snRNAseq dataset (Figure 2D). Hierarchical clustering of their expression shows that the differentially expressed genes are typically not expressed in all cell types, but rather the vast majority are observed to have much higher expression in 1 cell type.

475 (14.75%) and 327 (10.15%) of the differentially expressed genes have the highest average expression in the fast and slow myofibers, respectively. Considering that the VL has a higher proportion of fast myofibers than TA, we expected to observe many genes that are higher in VL from the bulk RNA analysis to be higher because they are expressed in fast fibers, and those higher in TA to be highest expressed in slow fibers. In line with this, we observed that differentially expressed genes that are higher in VL are more often expressed highest in fast fibers (445 genes, or 80.0% of the 556 genes higher in VL with highest expression in the myofibers) and conversely, differentially expressed genes that are higher in TA are most often restricted in their expression in slow fibers (216 or 87.8% of the 246 genes higher in TA with highest expression in the myofibers). However, there are exceptions to this expected pattern of expression based on the higher proportion of fast fibers in VL compared to TA (Supplementary Figure S1), and these may indicate shifts in metabolic phenotype within each muscle type. For instance, 111 of 1,559 genes higher in VL are most highly expressed in slow fibers (Supplementary Figure S1A) and 30 of 1,851 genes higher in TA have highest expression in fast fibers (Supplementary Figure S1B). Interesting exceptions include *CAMK2A* (encoding CaMKIIα) which is among the genes higher in the VL with higher expression in slow fibers than fast fibers. Conversely, *IRX3,* encoding iroquois homeobox 3, which has been linked to body weight (Gholamalizadeh et al., 2019), has ten-fold higher expression in fast fibers compared to slow fibers, but is higher in the TA muscles.

Remarkably, the remaining 75.1% of the differentially expressed genes have highest expression in other muscle resident cell types that are not the myofibers. Despite satellite cells, endothelial, smooth muscle and fibroblasts accounting for 6.60%, 7.01%, 0.69% and 3.17% of total cell population in both VL and TA, the percentage of differentially expressed genes with highest expression in these cell types were 15.71%, 14.96%, 10.87% and 8.20%, respectively, demonstrating differences in virtually all cells between skeletal muscle groups. Pericytes accounted for 17.57% and immune cells for 15.79% of all cells but had fewer genes that were detected as differentially expressed, 9.87% and 15.49%, respectively.

**FIGURE 3:**
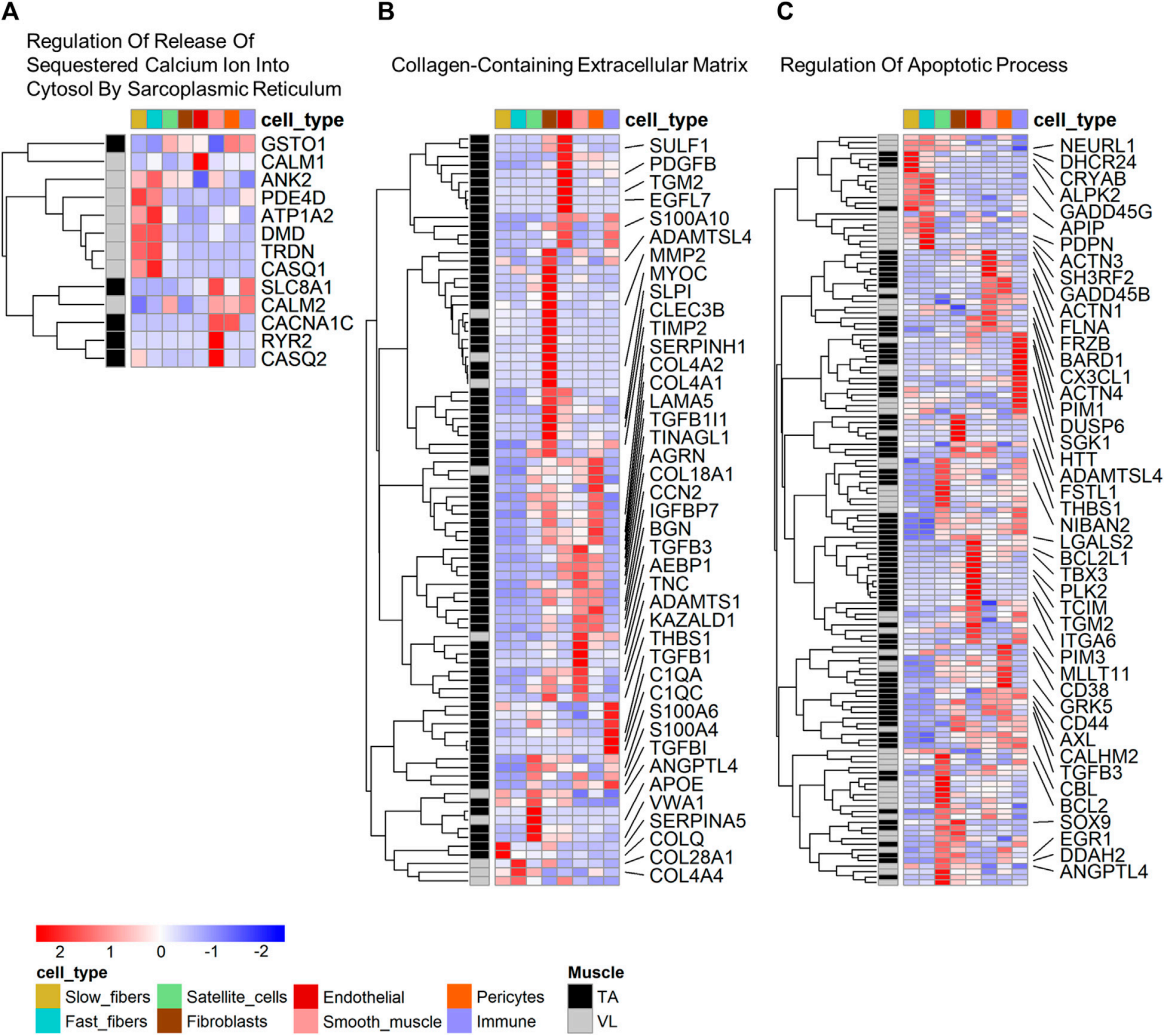
Cell type expression of genes within enriched gene ontology categories. Heatmap of expression of genes that are both differentially expressed and identified within select gene ontology categories shown in Table 1: **(A)** Regulation Of Release Of Sequestered Calcium Ion Into Cytosol By Sarcoplasmic Reticulum, **(B)** Collagen-Containing Extracellular Matrix, and **(C)** Regulation Of Apoptotic Process. For B and C, only select genes with a fold difference above 1.3 and average TPM in the muscle where it has highest expression above 5 are named. All data are present within Supplementary Tables S2A, B.

To determine which cell types have the highest expression of the differentially expressed genes within functional categories, we mapped cell expression using the single nuclei data (Supplementary Table S1). Eight of 13 (61.54%) genes in “Regulation Of Release Of Sequestered Calcium Ion Into Cytosol By Sarcoplasmic Reticulum” (Figure 3A) were higher in the VL, and these have highest expression predominantly in fast fibers (5 genes, 38.46%). These include *CALM1* and *CASQ1*, encoding calmodulin 1 and calsequestrin 1, respectively. Only *PDE4D* has highest expression in slow fibers, which we infer is differentially expressed independent of the differences in fiber type composition.

Among the genes in “Collagen-Containing Extracellular Matrix”, 78 of 88 (88.64%) genes have highest expression in the TA. Genes in this category have mapped highest expression in fibroblasts (26 genes, 29.55%), smooth muscle (14 genes, 15.91%), endothelial (13 genes, 14.77%) and pericytes (13 genes, 14.77%) (Figure 3B). “Collagen-Containing Extracellular Matrix” genes include metalloproteinase-2 (*MMP2*) which is responsible for remodeling the muscle extracellular matrix, a process important for proper satellite cell migration and differentiation (Chen and Li, 2009), along with tissue inhibitors of metalloproteinases, such as *TIMP*1 and *TIMP3*. Only 2 of the genes higher in TA (2.56%) have highest expression in myofibers, specifically in slow fibers (Figure 3B).

Genes in “Regulation Of Apoptotic Process” are more broadly expressed across all cell types (Figure 3C), suggesting that differential regulation of cell death is a characteristic of all cells in the VL and TA due to muscle origin. 22 genes have highest expression in the myofibers, including the heat shock protein *CRYAB* higher in the TA, mapping to the slow fibers and also annotated in the biological process category “Negative Regulation of Apoptotic Process”, which is also enriched among the differentially expressed genes (Supplementary Table S2A). Among genes with higher expression in other muscle resident cells, the widely studied anti-apoptotic BCL-xL/*BCL2L1*, higher in the TA, was highest expressed in endothelial cells. Despite not being annotated in “Regulation Of Apoptotic Process”, Hzf (encoded by *ZNF385A*) has been linked to negative regulation of apoptosis. In conditions of DNA-damaging stress, Hzf induction and binding to p53 modulates p53-mediated transcription such that the expression of pro-arrest p53 target genes is preferentially activated over pro-apoptotic p53 target genes (Das et al., 2007). *ZNF385A* is the most statistically significant gene with a 4.2X higher expression in TA (*p* = 1.76E-102) (Figure 1D), and has the highest expression in pericytes (average expression 0.31 TP10K) and similar levels of expression in fast and slow fibers (average expression 0.03 TP10K in both fiber types). These data suggest that the VL and TA have differential regulation of apoptotic signaling, with a potentially superior negative regulation in the TA that may be protective in DMD.

### 3.4 Search for transcription factors that may underlie the differential gene expression

Using the ENCODE_and_ChEA_Consensus_TFs_from_ChIP-X enrichment category within EnrichR (Chen et al., 2013), we identified 45 transcription factor genes that are reported to bind to multiple differentially expressed genes between VL and TA, and these may thus regulate the differentially expressed genes (Supplementary Table S2D). Because some transcription factors can act as both positive and negative regulators of expression, we searched for transcription factors that bind upstream of all differentially expressed genes, independently of the muscle in which they are highest expressed. Among these 45 transcription factors, 38 were expressed in the VL/TA bulk RNAseq dataset, and 11 were differentially expressed. The top 6 by *p*-value are: *TP63*, *AR*, *GATA2*, *KLF4*, *SMC3*, and *SMAD4* (Supplementary Table S2D). For each transcription factor, the putative target genes are listed in Supplementary Table S2D. These genes are further categorized in “Regulation Of Release Of Sequestered Calcium Ion Into Cytosol By Sarcoplasmic Reticulum”, “Collagen-Containing Extracellular Matrix”, and “Regulation Of Apoptotic Process” by the muscle in which they are highest expressed and listed in descending fold difference between the muscles (Supplementary Table S2D).

The 11 differentially expressed transcription factors were detected by snRNAseq (Supplementary Figure S2). The transcription factors higher in TA (*ZMIZ1*, *FOSL2*, *GATA2*, *KLF4* and *EGR1*) are mainly expressed in non-myolineage cell types, and mainly in fibroblasts and endothelial cells, consistent with a potential role regulating the extracellular matrix gene expression in non-myolineage cells. Among the differentially expressed genes in “Collagen-Containing Extracellular Matrix” and higher in TA, the metalloprotease *MMP2* is a target of GATA2 (Supplementary Table S2D). The transcription factors higher in VL are expressed in myolineage and non-myolineage cell types. *TP63* is restricted to the myofibers, and highest in fast fibers, whereas *AR* is highest expressed from satellite cells. Among the differentially expressed genes in “Regulation Of Release Of Sequestered Calcium Ion Into Cytosol By Sarcoplasmic Reticulum” is the TP63 target *ATP1A2* (Supplementary Table S2D), highest expressed in fast fibers (Supplementary Table S1), and the AR target *CALM1* (Supplementary Table S2D) with highest expression in endothelial and fast fibers (Supplementary Table S1).

### 3.5 Genes that are previously reported as DMD biomarkers and genetic modifiers are enriched among genes differentially expressed between healthy VL and TA

To explore potential relationships between our differential gene expression and reported mechanisms of DMD pathology that can be mapped to individual genes, we analyzed the differential expression of previously reported human serum/blood DMD biomarkers (Hathout et al., 2014; Parolo et al., 2018; Spitali et al., 2018; Al-Khalili Szigyarto, 2020; Grounds et al., 2020; Alonso-Jiménez et al., 2021; Wagner et al., 2021; Lee-Gannon et al., 2022; Wu et al., 2022), and of genes modifying the phenotype of DMD in humans (Pegoraro et al., 2011; Flanigan et al., 2013; Bello et al., 2016; Hogarth et al., 2017; Li et al., 2018; Weiss et al., 2018; Spitali et al., 2020; Flanigan et al., 2023) or the *mdx* mouse (Deconinck et al., 1997; Wagner et al., 2002; Han et al., 2011; Morales et al., 2013; de Zélicourt et al., 2022), also known as genetic modifiers, across the VL and TA and identified the predominant cell type in which they were expressed.

Among 88 DMD biomarkers expressed in healthy muscle, 41 (46.59%) were differentially expressed between VL and TA (Supplementary Figure S3A), a significant enrichment of this set of genes among differentially expressed genes (only 13 expected, *p*-value < 1E0-5, χ^2^ test). Of this set of differentially expressed biomarkers, 20 of 41 (48.78%) have highest expression in the myofibers, with 10 of these being more expressed in the slow and 10 in the fast fibers. Among these myofiber-derived biomarkers, 13 have the highest expression in VL, and 9 of these (69.2%) have highest expression in the fast fibers (*ALDOA*, *CAMK2B*, *ENO3*, *GAPDH*, *LDHA*, *MSTN*, *MYL1*, *PYGM*, *TNNI2*). The remaining 4 biomarkers higher in VL have either highest expression in the slow fibers (*CAMK2A*, *ACTA1*) or are similarly expressed in fast and slow fibers (*CKM*, *TTN*).

We also culled from the literature 18 genes described as genetic modifiers based on either a human genetic variant association with a DMD phenotype (*SPP1*, *ACTN3*, *THBS1*, *LTBP4*, *HLA-A*, *DYNLT5*, *CD40*, *NCALD*, *ETAA1*, *ADAMTS19*, *MAN1A1*, *GALNTL6*, *PARD6G*) or an *mdx* phenotype modified by concomitant deletion of another gene (*MSTN*, *DYSF*, *CCN2*, *UTRN*, *CD38*) (Deconinck et al., 1997; Wagner et al., 2002; Han et al., 2011; Pegoraro et al., 2011; Flanigan et al., 2013; Morales et al., 2013; Bello et al., 2016; Hogarth et al., 2017; Li et al., 2018; Weiss et al., 2018; Spitali et al., 2020; de Zélicourt et al., 2022; Flanigan et al., 2023). All were observable within the RNAseq and snRNAseq datasets, except *SPP1* (encoding osteopontin), which has a median TPM of 0.01 across VL and TA RNAseq and was not detected in snRNAseq of healthy VL and TA in any cell type. Thus, *SPP1* was below the limits of detection, and it was excluded from the differential gene expression analysis. 11 of 17 (64.7%) remaining DMD genetic modifiers were differentially expressed between VL and TA (Supplementary Figure S3B). This is a larger number than expected from a random sampling of all genes (only 3 expected, *p*-value < 1E0-5, χ^2^ test). Of these 11 genetic modifiers with differential expression detected, *DYSF*, *MSTN*, *ACTN3*, *NCALD*, *ADAMTS19* and *CD38* were higher in the VL, and *HLA-A*, *UTRN*, *LTBP4*, *CCN2*/CTGF, and *THBS1* were higher in the TA (Supplementary Figure S3B). This indirectly supports the relevance of genetic variants indeed contributing to differential disease progression across individuals. Most of the genetic modifiers (10 of 17) had expression mainly within a non-myofiber cell type, consistent with the known role of non-myofiber lineage cells in orchestrating muscle remodeling during regeneration and fibrosis (Mann et al., 2011).

*LTBP4* is most expressed in fibroblasts (Supplementary Figure S3B), consistent with prior reports on its ameliorative effect through a reduction in TGF-β signaling in fibroblasts with the IAAM haplotype (Flanigan et al., 2013). In addition to fibroblasts, *LTBP4* also shows high expression in satellite cells, suggesting the potential of a modifying effect acting upon muscle stem cells that has not been studied previously. *DYNLT5* (also known as *TCTEX1D1*) has a median TPM of 0.6 in the bulk RNAseq dataset, and it was not detected in fast or slow fibers by snRNAseq but was rather expressed in endothelial cells. This suggests its modifying mechanism is not due to direct expression within myofibers, or that it could be upregulated in a cell type other than endothelial cells in DMD to exert its modifying mechanism. *NCALD*, encoding the calcium-sensing neurocalcin delta, is most expressed in smooth muscle (7.11 TP10K), and has lower expression in fast (1.90 TP10K) and slow (0.42 TP10K) fibers. Its proposed mechanism is via regulation of a surrogate cGMP pathway that compensates for the defective nitric oxide-induced cGMP production in DMD, with lower expression of *NCALD* being protective (Flanigan et al., 2023). Consistent with this proposed mechanism, *NCALD* is not only higher in the VL in bulk RNAseq, but also in the VL fast (2.51X, *p* = 2.11E-76) and VL slow (1.11X, *p* = 3.01E-02) fibers compared to the TA fast and slow fibers, respectively. *HLA-A* is expressed higher in the VL, and class I MHC expression on myofibers may influence immune mediated mechanisms of myofiber damage in dystrophic muscle.

Only four reported genetic modifiers, *DYSF*, *ADAMTS19*, *MSTN*, and *ACTN3* are observed to have the highest expression in myofibers (Supplementary Figure S3C). The expression pattern of *DYSF*, *MSTN* and *ACTN3* is consistent with their described modifying mechanisms (Wagner et al., 2002; Vincent et al., 2007; Han et al., 2011). *DYSF* has similar expression in fast and slow fibers, with a slight 1.2X higher expression in fast fibers. Although the proposed modifying mechanism of *ADAMTS19* is through extracellular matrix (ECM) remodeling and TGF-β signaling (Flanigan et al., 2023), its highest expression in healthy muscle was not in fibroblasts (0.12 TP10K) or vasculature cells that typically produce ECM, but rather in the fast (1.58 TP10K) and slow (1.07 TP10K) fibers. A 13.2X higher expression of *ADAMTS19* in the fast fibers compared to fibroblasts suggests a modifying role in the myofibers that needs further exploration. At the single cell level, *ADAMTS19* is 1.55X higher in the VL slow fibers compared to the TA slow fibers (*p* = 1.18E-14), which further contributes to its higher expression in the VL. *MSTN* is expressed in both fast and slow fibers, with highest expression in fast fibers (4.4X compared to slow fibers), a pattern of expression that contributes to it being higher in VL by bulk RNAseq, as VL has a higher proportion of fast fibers. *ACTN3* is highly specific to fast fibers (13.3X higher in fast fibers), although not absent in slow fibers. In addition, at the single cell level, *ACTN3* expression is 1.43X higher in the VL fast fibers compared to the TA fast fibers (*p* = 2.17E-11), indicating that the higher expression of *ACTN3* in VL is influenced by both a higher proportion of fast fibers and by a VL-specific upregulation within the fast fibers.

**FIGURE 4:**
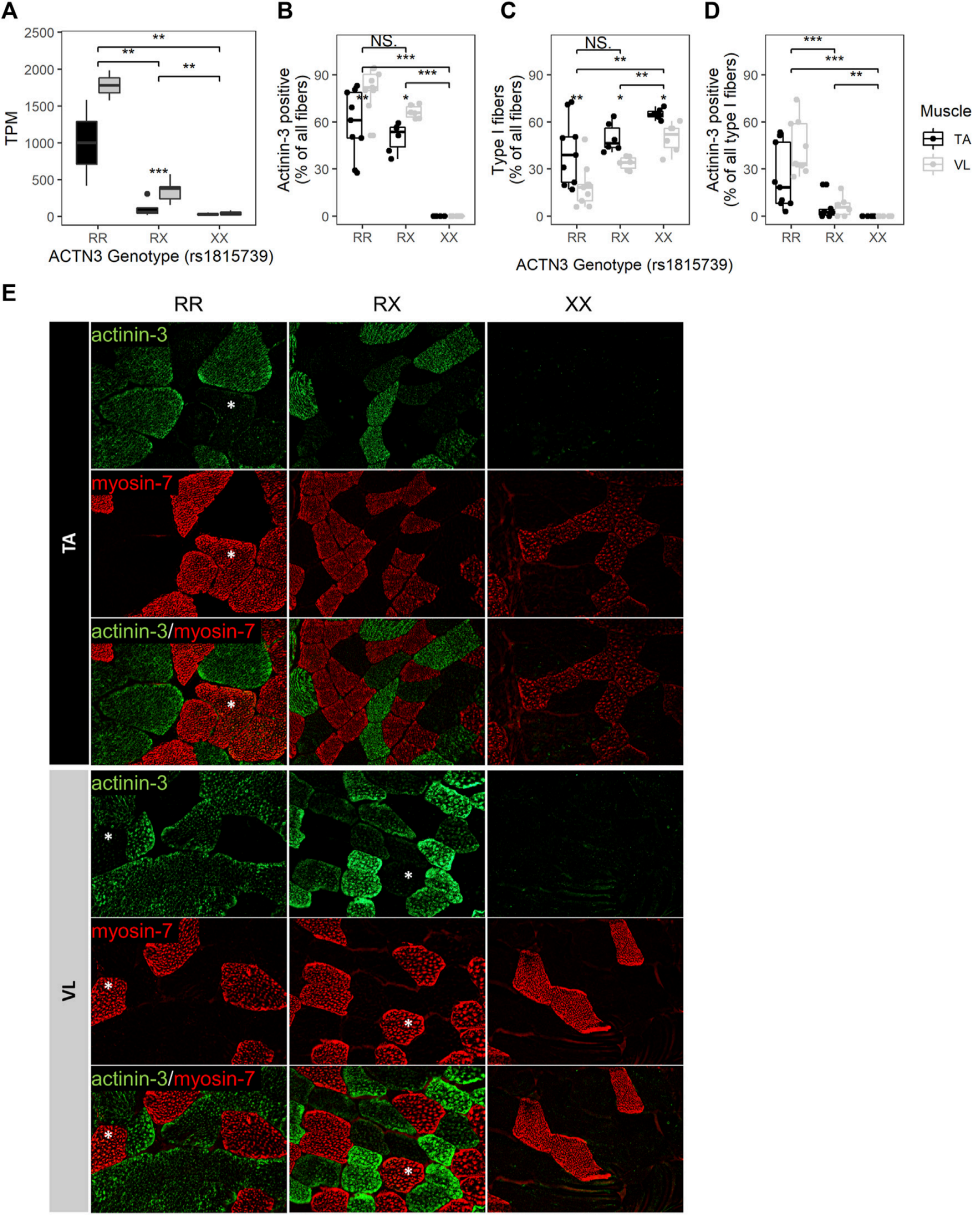
*ACTN3* genotype correlates with the proportion of slow fibers and with the expression of actinin-3 in slow fibers. **(A)** *ACTN3* TPM by genotype at the rs1815739 polymorphism locus (NM_001104.4:c.1729C>T, NP_001095.2:p.Arg577Ter/p.R577X) for all 27 samples in the bulk RNAseq dataset (RR *n* = 2, RX = *n* = 9, XX *n* = 3). Percentage of all counted fibers that are actinin-3 positive **(B)** and type I (slow) **(C)**. Percentage of all type I fibers that are actinin-3 positive **(D)**. Wilcoxon test, *: p ≤ 0.05, **: p ≤ 0.01, ***: p ≤ 0.001. **(E)** Representative images of actinin-3 and myosin-7 staining. Magnification = 20X. Asterisks indicate actinin-3 positive type I fibers. The bars in the box plots indicate 1.5* IQR, which is the interquartile range, or the distance between the first and third quartiles.

The well-studied null allele of *ACTN3* (rs1815739, NM_001104.4:c.1729C>T, NP_001095.2:p.Arg577Ter/p.R577X) is a common allele found in the population with a frequency of the X allele of 0.36 (dbSNP). Actinin-3 loss was associated with a reduced DMD severity as measured by a longer 10-min walk test (Hogarth et al., 2017), and this was attributed to a switch to a more protective oxidative metabolism without a shift in fiber type distribution (MacArthur et al., 2008). To further investigate the effects of *ACTN3* expression across muscles, we genotyped rs1815739 in the 15 individuals. We identified 3 null homozygotes (XX), and 3 reference homozygotes (RR), and 9 heterozygotes (RX) among the 15 individuals. The expression of *ACTN3* was significantly differentially expressed dependent on genotype (Kruskal–Wallis *p* = 7.73E-04), with the RR group showing highest expression, indicating nonsense-mediated decay of the X allele (Figure 4A). XX homozygotes have no *ACTN3* mRNA expression for both VL and TA muscles (Figure 4A). The expression of *ACTN3* RR and RX mRNA was consistently higher in the VL compared to TA, although only statistically significant in the RX genotype (Wilcoxon *p* = 9.9E-04). For both the VL and TA, the mean level of *ACTN3* mRNA in RX heterozygotes was substantially lower than the expected 50% of the RR genotype mRNA level, suggesting that the X allele reduces the expression of the R allele through unknown mechanisms.

To validate the RNA findings and assess whether the *ACTN3* genotype groups have differences in fiber type composition, we performed immunofluorescent staining for 3 RR, 2 RX and 2 XX individuals’ VL and TA. For each sample and muscle, 3 10 µM tissue sections were stained (total of 42 sections), and fibers were counted across the entire sections. An average of 204 fibers were counted per sample (range 25–758, total 8,577) (Supplementary Figure S4). Observed differences in mRNA expression were consistent with antibody staining for actinin-3. As expected, *ACTN3* XX homozygotes showed no detectable protein (Figures 4B,E). The percentage of actinin-3 positive fibers was consistently higher in the VL for both RR and RX genotypes (Figure 4B). The percentage of type I slow fibers (positive for myosin-7) was consistently higher in the TA across genotype groups (Figure 4C), as expected (Edgerton et al., 1975; Jakobsson et al., 1991). There was a higher percentage of type I slow fibers in the RX and XX groups compared to the RR group across both muscles, although only the XX group reached statistical significance (Figure 4C). There was also a higher percentage of type I fibers in the XX compared to RX group. These findings are supported by observed similar relative expression of the myosin heavy chain genes at the RNA level (Supplementary Figure S5). Interestingly, we also identified slow fibers with low expression of actinin-3 (Figure 4D) in the RR and RX genotypes but not in the XX genotypes, reflective of a low level of expression of actinin-3 in some slow myofibers. This low level of expression is only apparent because of the true null staining revealed in the XX genotype individuals.

### 3.6 Identification of druggable targets within differentially expressed genes

We place the list of differentially expressed genes into context as potential for disease modification because their RNA or protein products are targeted or ‘druggable’ with existing drugs documented in the DrugBank database (Wishart et al., 2018). 535 of 3,410 (15.7%) differentially expressed genes are reported targets of 1,812 known drugs (Supplementary Table S1). The protein product of 197 genes higher in the VL are targeted by 984 drugs, and thus may constitute a set of known drugs that may be explored to induce a shift of a VL-like susceptible state towards a TA-like protected state in DMD. Druggable genes expressed higher in VL are enriched in 158 pathways (Supplementary Table S2E). Among the top 5 most significant pathways is calcium signaling pathway, with 22 genes higher in VL that include calmodulin (*CALM1*, *CALM2*), calmodulin-dependent kinases (*CAMK2A*, *CAMK2B*, *CAMK2G*), calsequestrin (*CASQ1*), ryanodine receptor (*RYR1*), and the dihydropyridine receptor alpha 1S subunit (*CACNA1S*). These 8 genes alone are reported to be targeted by 71 drugs and may suggest ways to therapeutically regulate intracellular and sarcoplasmic reticulum (SR) calcium concentration in myofibers. *CD38* (1.7X higher in VL) is highest expressed in the pericytes (1.83 TP10K), but also is expressed in fast (1.28 TP10K) and slow (0.48 TP10K) fibers. Its higher expression in VL is also observed at the single cell level, with 1.24X higher expression in VL fast fibers compared to TA fast fibers (*p* = 4.71E-04). *CD38* encodes a NAD+ glycohydrolase that produces regulators of Ca2+ signaling, and deletion of *CD38* or treatment with CD38 inhibitors restored the *mdx* heart, diaphragm and limb function, reduced fibrosis and inflammation, and reduced the cycles of degeneration and regeneration (de Zélicourt et al., 2022). DMD myotubes treated with a monoclonal antibody against CD38 (Isatuximab) reduced the frequency of spontaneous Ca2+ waves (de Zélicourt et al., 2022).

### 3.7 Identification of isoforms with differential abundance between VL and TA

**FIGURE 5:**
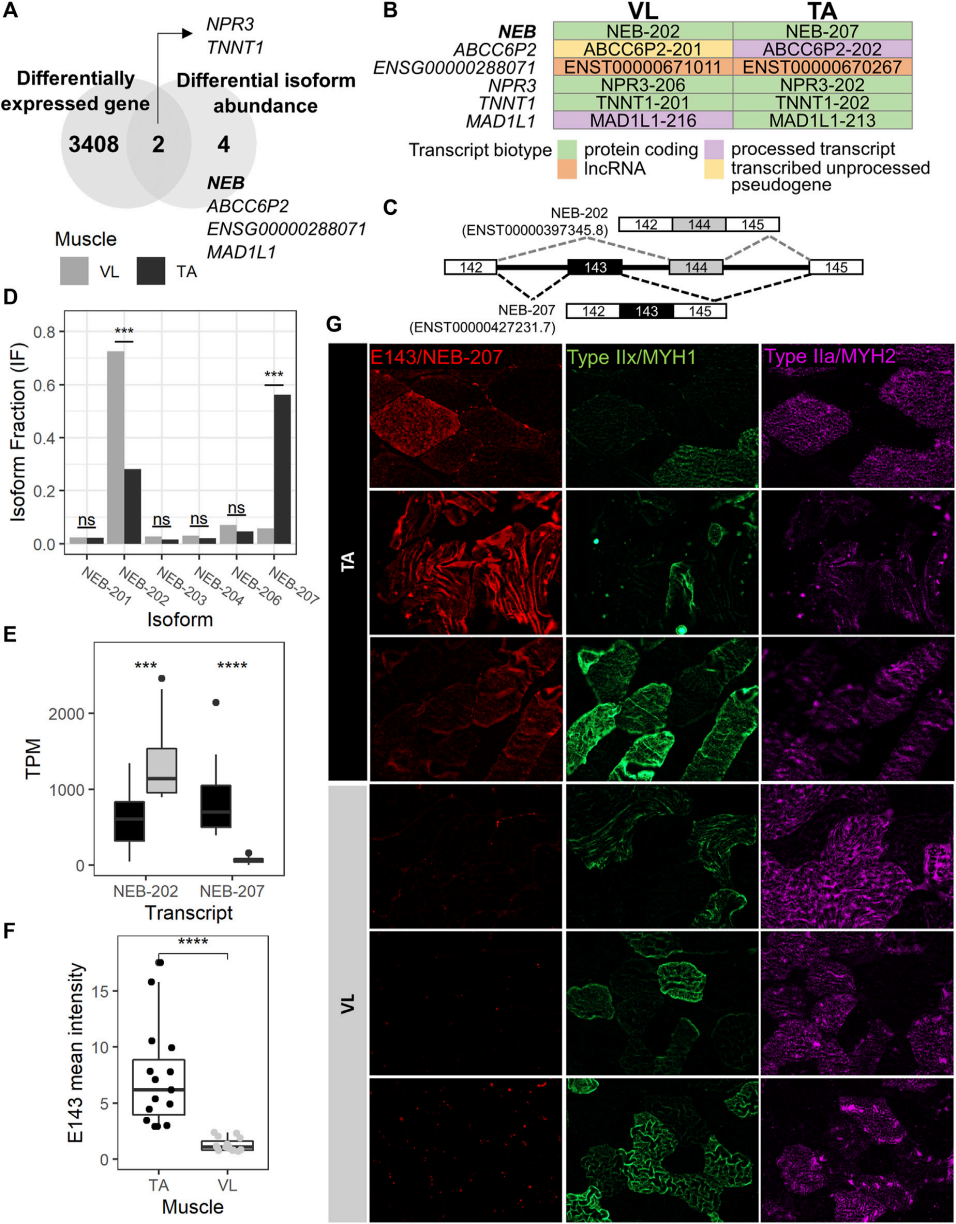
NEB-207 is upregulated in the TA across all fiber types. **(A)** Venn diagram showing the overlap of differentially expressed genes and genes with differential isoform abundance (isoform switch) between VL and TA. **(B)** For each muscle, the Ensembl transcript biotype of each preferentially used isoform is shown for each isoform switch event. **(C)** Diagram of the alternative usage of exons 143 and 144 in *NEB*. The resulting isoform name is indicated for each exon usage, and corresponding transcript IDs are in parenthesis. **(D)** Isoform fraction (usage) of expressed *NEB* isoforms obtained from IsoformSwitchAnalyzeR. **(E)** Expression of the NEB-202 and NEB-207 isoforms obtained from the Kallisto alignment. TPM = Transcripts per million. **(F)** Quantification of the overall mean immunofluorescence signal intensity of nebulin exon 143 (NEB E143) in 5 20X images for each VL and TA among 3 healthy individuals. Wilcoxon test *p* = 1.29E-08; ****: p≤0.0001). **(G)** Representative images of immunofluorescence staining of paired VL and TA sections.

Because extensive alternative splicing is observed in developing and mature muscle (Brinegar et al., 2017; Nakka et al., 2018), including in genes encoding sarcomere structural (Donner et al., 2004; Bowman et al., 2007; Lam et al., 2018; Savarese et al., 2018), and excitation-contraction coupling proteins (Nakka et al., 2018), we hypothesized that the VL and TA transcriptomes are also differentially influenced by alternative splicing leading to significant shifts in the usage of specific isoforms (isoform switch). To identify isoform switching events between VL and TA, we utilized IsoformSwitchAnalyzeR (Vitting-Seerup and Sandelin, 2017), which uses the abundance (TPM) and count data obtained from Kallisto transcript alignment. Prefiltering of the annotated transcripts resulted in 130,664 transcripts to be considered for isoform switch analysis. Further filtering of transcripts for switching genes with at least two significantly switching isoforms and with at least two isoforms preferentially used in opposed directions (higher in one muscle, lower in the other) resulted in 47 transcripts. Among these, 12 transcripts have a significant isoform switch (isoform switch q-value <0.05) between VL and TA, and these are located within 6 genes (gene switch q-value <0.05) (Supplementary Table S2F). Two of the 6 genes with isoform switching, *NPR3* and *TNNT1*, were differentially expressed between VL and TA, whereas the remaining 4 (*NEB*, *ABCC6P2*, *ENSG00000288071* and *MAD1L1*) did not have differential expression at the gene expression level (Figure 5A; Supplementary Table S1). *NEB* is highly and similarly expressed in slow (217.6 TP10K) and fast (185.4 TP10K) myofibers (Supplementary Table S1). *ENSG00000288071* and *TNNT1* are more highly expressed in the slow fibers (6.8X and 16.4X higher expression in the slow compared to fast fibers), and *ABCC6P2* in the fast fibers (0.009 TP10K in fast and not detected in slow fibers) (Supplementary Table S1). *NPR3* and *MAD1L1* are expressed highest in the smooth muscle cells (Supplementary Table S1). Four of 6 genes with isoform switch are protein coding (*NEB*, *NPR3*, *TNNT1* and *MAD1L1*), whereas *ENSG00000288071* is a long non-coding RNA, and *ABCC6P2* is a transcribed unprocessed pseudogene (Supplementary Table S1).

To assess potential functional consequences of the isoform switches, we examined the biotype of each isoform in the switching (Figure 5B). The *NEB*, *TNNT1* and *NPR3* isoform switches are among protein coding transcripts, and that of *ENSG00000288071* is among long noncoding RNAs (lncRNA). The remaining 2 genes (*ABCC6P2* and *MAD1L1*) are switching between isoforms of different biotypes (Figure 5B). ABCC6P2-202, preferentially used in TA, is a processed transcript, whereas ABCC6P2-201, preferentially used in VL, is a transcribed unprocessed pseudogene. MAD1L1-213, with preferential usage in TA, is protein coding, whereas MAD1L1-216, preferentially used in VL, is a processed transcript, which means that it does not have an open reading frame (a start codon followed by an in-frame stop codon (Kute et al., 2022)).

The *MAD1L1* isoform switch comprises MAD1L1-213 and MAD1L1-216 with the former used more in TA (absolute difference isoform fraction = 0.073) and the latter in VL (absolute difference isoform fraction = 0.043) (Supplementary Table S2F). *MAD1L1* has a relatively low expression in muscle. The top 3 isoforms expressed in both VL and TA have an average TPM ranging from 0.80 to 1.65. *MAD1L1* encodes for the mitotic arrest deficient-like protein 1 (also known as MAD1). In *mdx*, *Mad1l1* is most expressed in late activated satellite cells, myoblasts and myocytes (Scripture-Adams et al., 2022) (data not shown), suggesting a potential role in muscle regeneration in wild-type muscle and in DMD, although which isoform is most important is unknown. The *MAD1L1* isoform switch involves a switch from a protein coding isoform in TA to a processed transcript that has no open reading frame in VL, suggesting a potential mechanism of reducing its protein expression in the VL via alternative splicing, an event that cannot be detected by gene expression analysis.

The gene with the most striking isoform switch is *NEB*. This switch comprises the mutually exclusive exon splicing event that occurs between exons 143 and 144 of *NEB*, which has been previously described (Donner et al., 2004; Lam et al., 2018). Exons 143 (E143, included in NEB-207) and 144 (E144, included in NEB-202) are mutually exclusive exons (Figure 5C), such that in the same transcript, only one of either exon is included. NEB-207 has a higher usage in TA, a 0.505 isoform fraction difference compared to VL (Supplementary Table S2F; Figure 5D). NEB-202 has a higher usage in VL, with a 0.443 isoform fraction difference compared to TA (Supplementary Table S2; Figure 5D). This difference in isoform usage is readily observed at the isoform expression level (Figure 5E). NEB-207 has higher expression in TA, with an average TPM of 850, compared to an average TPM of 66 in VL. NEB-202 is more broadly expressed across both muscles but has a preferential expression in the VL with an average TPM of 1,357, compared to an average TPM of 641 in TA. We confirmed this differential alternative splicing event in the RNA by semi-quantitative reverse transcription polymerase chain reaction (RT-PCR) using exon junction-specific primers (data not shown).

Previous reports sought to determine whether the expression of nebulin exon 143 presents a fiber type-specific pattern in adult human quadriceps (Lam et al., 2018). In this previous study, E143 was found expressed more often in fast fibers compared to slow fibers (Lam et al., 2018), although a distinction between type IIa and type IIx fast fibers was not explored. Consequently, it was concluded that fast fibers usually express E143, and that slow fibers may express either E143 or E144. However, because we observe that the VL mainly includes E144 and not E143, and because VL has in average 1.82 more fast fibers than TA (Figure 2C), we reasoned that the differential pattern of expression of E143 between VL and TA is not solely dependent on fast fiber type. Thus, we assessed the protein expression of E143 across VL and TA in relation to type IIa and IIx fast myosin. We examined overall E143 protein intensity among VL and TA in 3 healthy individuals. We found low to no E143 myofiber intracellular protein expression in the VL among either fiber type (Figure 5G). Consistent with this observation, overall E143 protein intensity was statistically higher in the TA compared to the VL (Wilcoxon test *p* = 1.29E-08, 95% CI = 2.70-7.04, average fold difference = 5.77) (Figure 5F). These findings suggest that although nebulin including exon 143 is more often expressed in the fast fibers (Lam et al., 2018), and E143 is more consistently observed in the fast type IIx than in the slow type I (data not shown), a fast fiber type is not the sole determinant of its expression in human skeletal muscle. That is, that the association between fast myosin and exon 143 of nebulin, as described previously (Lam et al., 2018) is also muscle-type specific, and might be regulated by specific differentially expressed splicing factors, or their combinations.

To identify potential splicing factors underlying the alternative splicing observed between VL and TA, we looked for splicing factors that are differentially expressed between the muscles. Out of 66 expressed splicing factors obtained from the SpliceAid-F database (Giulietti et al., 2013), 18 (27.3%) were differentially expressed between VL and TA (Supplementary Table S1). Only one splicing factor, *NOVA2*, is higher in TA, and the remaining 17 are higher in VL, with *ESRP2* and *CELF2* being the most differentially expressed.

### 3.8 Comparison of VL *versus* TA differentially expressed genes with DMD *versus* healthy muscle differentially expressed genes

To assess whether the differentially expressed genes between healthy muscles differentially susceptible to lack of dystrophin are also changed in expression in the context of DMD, we generated bulk RNAseq data of the TA from 8 young ambulatory DMD patients (mean age 4.5 years). An average of 60 million paired end sequencing reads were generated per sample (range 43–72 million reads). To our knowledge, this is the second and largest reported bulk RNAseq dataset of DMD muscle (an existing dataset can be found in SRA ID PRJNA734152), and the first of the TA muscle.

**FIGURE 6:**
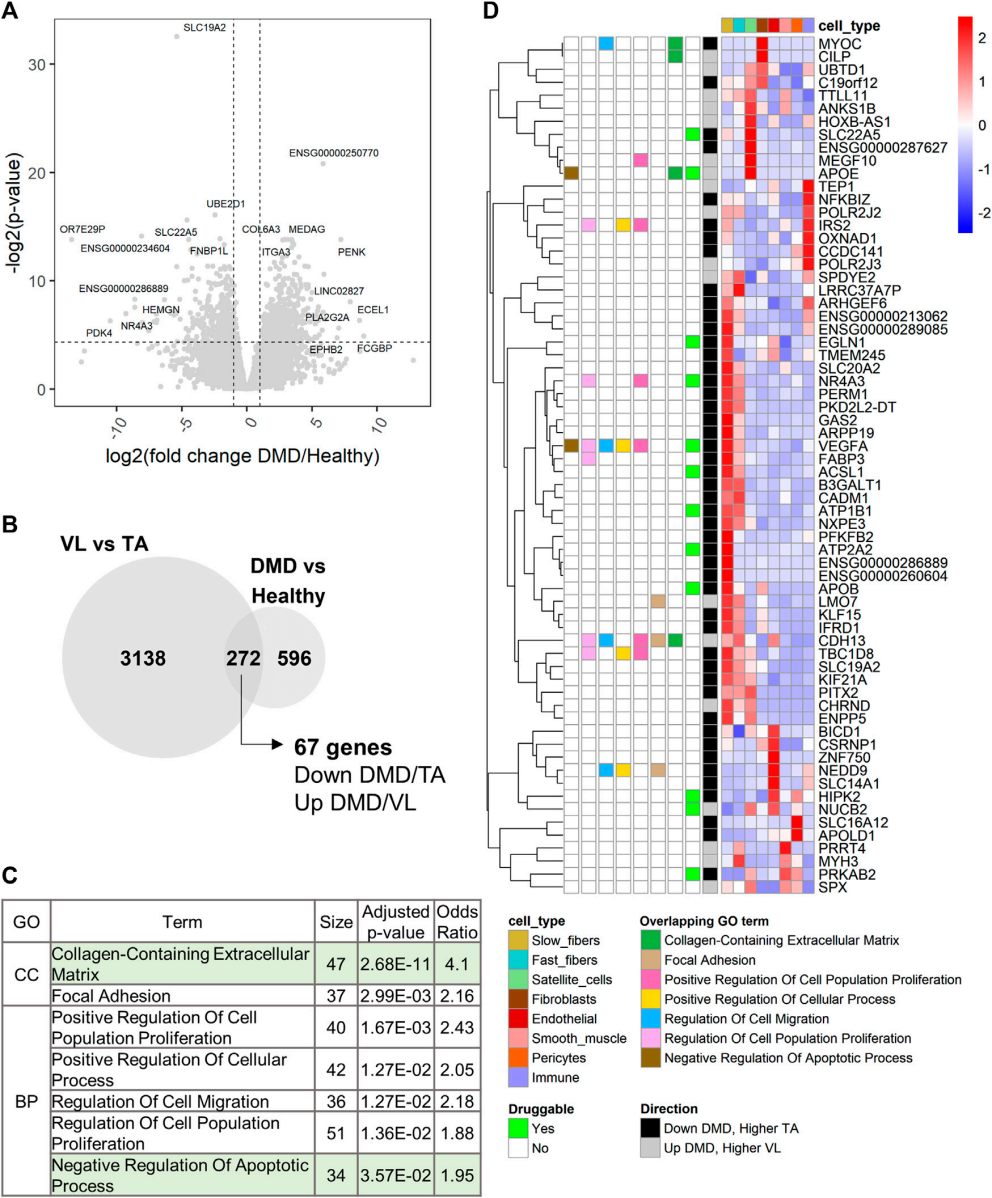
Extracellular matrix and regulation of apoptosis are also dysregulated in DMD. **(A)** Volcano plot for all 17,183 genes tested for differential expression between DMD and healthy TA. Dashed lines depict a fold change of 2 and a *p*-value of 0.05. For each up and downregulated genes, the top 5 differentially expressed genes by fold change and the top 5 genes by *p*-value are labeled. **(B)** Overlap of the differentially expressed genes between VL and TA and between DMD and Healthy. The 67 genes are those downregulated in DMD and higher in TA or upregulated in DMD and higher in VL. **(C)** 7 of 9 functional categories with over 30 genes that are shared between the two analyses (sorted by ascending *p*-value). “Nervous System Development” (which had only *VEGFA* in the 67-gene list, and “Cell-Substrate Junction” (which has the same 37 genes as “Focal Adhesion”, and a larger *p*-value) were excluded. **(D)** Single cell expression of the 67 overlapping genes. The overlapping GO terms from (C) in which each gene member is categorized among these 7 categories are indicated. “Druggable” indicates which genes have existing drugs documented on DrugBank. “Direction” indicates the direction of expression in both the VL *versus* TA and DMD *versus* Healthy differential gene expression analyses.

Using DESeq2 (Love et al., 2014), we performed differential gene expression analysis between DMD (*n* = 8) and healthy TA (*n* = 13). 868 of 17,183 analyzed genes were differentially expressed between DMD and healthy (Supplementary Table S3; Figure 6A). 272 of these genes were also differentially expressed between VL and TA (Figure 6B). Among these overlapping genes, 67 were downregulated in DMD and higher in the less susceptible TA or upregulated in DMD and higher in the more affected VL (Figure 6B). Next, we assessed functional enrichment in the genes dysregulated in DMD using EnrichR. 49 biological processes and 11 cellular component categories were enriched among genes dysregulated in DMD (adjusted *p*-value <0.05) (Supplementary Table S2G). Among these, 31 categories were also enriched among genes differentially expressed between VL and TA. “Collagen-Containing Extracellular Matrix” was the most significant shared term and also the most significant term among all enriched in DMD *versus* Healthy (Supplementary Table S2G). The categories with over 30 gene members include “Collagen-Containing Extracellular Matrix” and “Negative Regulation of Apoptotic Process”, supporting the involvement of these gene sets in both the differential susceptibility between VL and TA, and the dystrophic pathology (Figure 6C).

To identify candidate susceptibility factors that are also dysregulated in DMD, we further explored the 67 overlapping genes. All except one gene (*SBK3*) were detected in the healthy muscle snRNAseq (Figure 6D). 13 of these 67 genes are druggable (Figure 6D). Among the overlapping protein coding genes, the 3 most dysregulated genes in DMD (by fold change) are *NR4A3*, *APOB* and *MYOC*, and the 3 most differentially expressed in VL compared to TA are *APOB*, *SBK3* and *NR4A3*, highlighting the relevance of these genes in DMD and consequently, the differential susceptibility of VL and TA (Supplementary Table S3).

Among genes in “Collagen-Containing Extracellular Matrix” are *MYOC* and *CILP* (Figure 6D). *MYOC*, encoding myocilin, is most expressed in fibroblasts (Figure 6D; Supplementary Table S1) and is downregulated 47.5X in DMD (Supplementary Table S3), despite the expansion of fibroblasts within dystrophic muscle (Scripture-Adams et al., 2022), suggesting a downregulation in dystrophic fibroblasts. Myocilin has been widely studied in glaucoma as a secreted protein in the trabecular meshwork (Resch and Fautsch, 2009). Myocilin was also found to be induced during C2C12 myoblast differentiation via regulation of the TGF-β pathway (Zhang et al., 2021), and to interact with the dystrophin-glycoprotein complex via syntrophin (Joe et al., 2012). In the Human Protein Atlas (Uhlén et al., 2015), *MYOC* is highest expressed in fibroblasts in skeletal muscle, and not in myocytes, consistent with our observations. The overexpression of *MYOC* increases muscle mass (Joe et al., 2012), and downregulation of myocilin is observed in cancer cachexia, with its loss inducing muscle fiber atrophy and an increase in fibrotic and fatty tissue (Judge et al., 2020). Because its expression is highest in fibroblasts, and it is found within “Collagen-Containing Extracellular Matrix”, we hypothesize that its main role in human skeletal muscle is in the fibroblasts, and not the myolineage, and that its downregulation promotes fibrosis. These data, along with the 1.77X higher expression of *MYOC* in the TA (Supplementary Table S1), support myocilin as a protective factor for the TA.

Conversely, *CILP*, encoding the cartilage intermediate layer protein 1 (CILP-1), is upregulated 4.3X in DMD (Supplementary Table S3), and is 1.27X higher in the more susceptible VL. *CILP* also has highest expression in the fibroblasts in our dataset (Figure 6D; Supplementary Table S1) and in the Human Protein Atlas (Uhlén et al., 2015), but its role is not well understood. Upregulation of CILP-1 occurs upon cardiac injury in fibrotic regions, and there is a decrease in serum of patients with heart failure (Park et al., 2020). Its anti-fibrotic effect in pressure-overload cardiac remodeling (Zhang et al., 2018) suggests that CILP-1 is regulated in relation to processes that involve cardiac fibrosis. Because of its upregulation in DMD and higher expression in VL, and its restricted expression in the fibroblasts, we hypothesize that CILP-1 is pro-fibrotic in skeletal muscle, and a susceptibility factor for the VL.

Among other overlapping genes is *SLC19A2*, which is the most statistically significantly dysregulated gene in DMD compared to healthy TA (*p* = 1.59E-10), and which is downregulated 41.77X in DMD (Supplementary Table S3). *SLC19A2* encodes the thiamine (vitamin B1) transporter 1 (THT1), which has highest expression in skeletal muscle (GTEx), specifically in slow fibers (Figure 6A; Supplementary Table S1). Thiamine supplementation has been shown to improve muscle strength in myotonic dystrophy type 1 (Costantini et al., 2016). In *mdx*, supplementation with the thiamine precursor benfotiamine ameliorated the dystrophic pathology and increased grip strength (Woodman et al., 2018), supporting a protective role for the TA compared to VL in DMD, and potentially also in the slow fibers compared to fast fibers. Lastly, *KIF21A*, highest expressed in slow fibers, is downregulated 2.91X in DMD (Supplementary Table S3), and is 1.20X higher in the TA (Supplementary Table S1). Heterozygous mutations in *KIF21A* cause autosomal dominant congenital fibrosis of extraocular muscles (EOM) (Yamada et al., 2003). The downregulation of *KIF21A* in DMD skeletal muscle, reduced function leading to pathologic fibrosis in EOM, and its higher expression in the TA suggest a protective role of higher *KIF21A* expression within myofibers that leads to some protection from damage in DMD. *KIF21A* encodes a kinesin, involved in cargo transport between the Golgi apparatus and the endoplasmic reticulum (Hirokawa and Noda, 2008), but its role in skeletal myofibers is not established.

Among the 67 overlapping genes, *APOE* is found in “Negative Regulation of Apoptotic Process” and is upregulated in DMD and higher in the VL. *APOE* is a highly specific satellite cell marker in healthy muscle (Figure 2B), suggesting that a potential differential regulation of apoptotic signaling (Figure 3C) may alter the regenerative capabilities of VL and TA.

Cell type specificity of the 67 genes differentially expressed between VL and TA and also dysregulated in DMD (Figure 6D) was examined within previously published single cell and nuclei RNAseq datasets from the mouse TA (scMuscle) (McKellar et al., 2021) and soleus (myoatlas) (Petrany et al., 2020). Using these datasets, the cell type specificity of 17 protein coding genes was confirmed. These include *MYOC* and *CILP*, highest expressed in the fibroblasts in both scMuscle (Supplementary Figure S6A) and myoatlas (Supplementary Figure S6B) in mouse.

We note *PDK4* as the most dysregulated gene within “Negative Regulation of Apoptotic Process”, and the most dysregulated protein coding gene among all 868 genes differentially expressed between DMD and healthy TA in our transcriptome-wide analysis. *PDK4*, encoding pyruvate dehydrogenase kinase 4, is downregulated 1,453X in DMD and only superseded by the unprocessed pseudogene *OR7E29P,* which is downregulated 11,396X (Figure 6A; Supplementary Table S3). Downregulation of *PDK4* in DMD has been previously reported in the slow (type I) fibers (1.74X, *p* = 2.78E-04), and upregulation in the fast type IIa and IIx (Scripture-Adams et al., 2022). *PDK4* is also downregulated in *mdx* quadriceps and TA compared to age-matched controls (Matsakas et al., 2013). *PDK4* is downregulated in DMD, but higher in the more susceptible VL (1.98X, Supplementary Table S1). Interestingly, *PDK4* is a druggable gene, with tretinoin (a vitamin A derivative) being a known upregulator (Supplementary Table S1).

Lastly, among the 17 known DMD genetic modifiers included in our DMD *versus* Healthy comparison, none were significantly dysregulated in DMD compared to Healthy TA (Supplementary Table S3), but some trended toward a significant upregulation after multiple testing correction (*p* < 0.30), including (by ascending *p*-value): *LTBP4* (4.47X, *p* = 5.37E-02), *MAN1A1* (4.01X, *p* = 6.21E-02), *THBS1* (24.89X, *p* = 9.76E-02), *PARD6G* (3.50X, 1.54E-01), *SPP1* (139X, *p* = 2.80E-01), and *NCALD* (2.39X, *p* = 2.97E-02). Interestingly, all 6 genes have variants that have been identified to modulate the disease progression in DMD patients (*versus* in *mdx* double knockouts), supporting their relevance in human pathology.

## 4 Discussion

Our goal in this study was to analyze two different healthy limb muscles with more similar functional roles (VL and TA), which have a consistently observed difference in disease progression, that is more modest in degree than the greater protection from damage of EOM compared to limb muscles in the absence of dystrophin. Because of published longitudinal imaging data (Rooney et al., 2020), we could select comparable muscles amenable to biopsy in healthy adults (Barthelemy et al., 2020). The protection of TA relative to VL is less striking than that of EOM compared to limb but is still substantial with an estimated shift in equivalent damage of 8.5 years in humans (Rooney et al., 2020). VL and TA demonstrate a substantial difference in their susceptibility to lack of dystrophin, and our transcriptomic study of paired samples from the same healthy individuals identifies a large portion of the transcriptome as altered, with 3,410 differentially expressed genes. There is inherent biological variability within each large muscle, and there is some potential variability added to transcriptomic comparisons due to the small sample analyzed, which may not be representative of the whole muscle. The relatively small sampling by biopsy can introduce variability from sampling different parts of each muscle. This could result in a reduction in the number of differentially expressed genes, but should not lead to false expression differences between muscle groups. We try to limit variation by sampling the same relative location of VL and TA, which indeed resulted in highly significant differential gene expression detection. Because the transcriptomic differences between muscles in this study greatly exceeded those driven by genetic variation, we note that future studies may not require a paired design approach that was used here to maximize discovery and control for interindividual differences.

Our study particularly highlights calcium homeostasis, ECM, and regulation of apoptosis, and provides a dataset for exploration to investigate potential protective mechanisms of myofibers to loss of dystrophin in skeletal muscle. By studying muscles that have a substantial difference in their rate of disease progression in DMD, we sought to identify mechanisms of myofiber protection, complement genetic modifier studies and reveal novel therapeutic targets. There is some overlap between prior gene expression work comparing EOM to limb muscles and this study comparing TA to VL, including enrichment of genes with functions related to sarcomere structure, calcium homeostasis, muscle development, metabolic and immune processes, vasculature development, regulation of cell death and extracellular matrix, and thus supports that these pathways are relevant to how muscles are differentially susceptible to damage with lack of dystrophin. Comparison with genes dysregulated in DMD skeletal muscle compared to healthy further highlights the potential role of ECM and negative regulation of apoptosis in the differential susceptibility of VL and TA in DMD. We note that the mean age of our DMD cohort (4.5 years) is younger than the healthy cohort (21.2 years), and we attempted to reduce the effect of age on the identified gene expression differences. However, age could also be contributing to gene expression differences reported here.

Recently, a relatively higher regenerative capacity of EOM muscle stem cells was identified and attributed to upregulation of thyroid-stimulating hormone receptor (TSHR) signaling through upregulation of adenylate cyclase activity in EOM relative to limb muscle (Taglietti et al., 2023). Although *TSHR* is not differentially expressed between VL and TA in our study, “Adenylate Cyclase-Activating G Protein-Coupled Receptor Signaling Pathway” trended toward significance among the biological process GO terms (*p* = 9.92E-02), with 16 of the 17 genes higher in the TA (data not shown), suggesting a protective role in the TA and consistent with the proposed therapeutic relevance of upregulation of adenylate cyclase in DMD, where adenylate cyclase activation stimulates TSHR signaling, reduces muscle stem cell senescence and improves their proliferation (Taglietti et al., 2023).

Of note, only 24.9% of the differentially expressed genes were highest expressed in the myofibers, indicating that many of non-myofiber cells are likely to play an important role in protecting myofibers from death. A caution of our work is that the healthy muscles are sampled without active degeneration/regeneration or induced muscle damage, which is a chronic state in DMD, and thus our data does not necessarily reveal mechanisms that may be only induced with muscle injury.

ECM deposition is an important component of the muscle structure and function (Loreti and Sacco, 2022), and there is an enrichment of differentially expressed genes that encode “Collagen-Containing Extracellular Matrix”. ECM remodeling is necessary to properly activate muscle stem cells during regeneration, and the dysregulation of ECM proteins has been associated with regeneration defects in muscle diseases (Loreti and Sacco, 2022). In addition, the ECM stiffness, which varies depending on ECM composition, can modulate satellite cell activity and myofiber-generated force during contraction, and undergoes changes with age (Sinha et al., 2020). Thus, observed differences in ECM gene expression in VL and TA may contribute to their differential progression in DMD and cause differences in the fibrotic response within each muscle type. We highlight *MYOC* as a potentially protective anti-fibrotic, and *CILP* as a potentially damaging pro-fibrotic gene in DMD.

Myofiber death in DMD has been mainly attributed to necrosis (Bencze, 2023). However, a higher rate of apoptotic nuclei in DMD compared to healthy muscle has been repeatedly observed (Tews and Goebel, 1997; Sandri et al., 1998; Serdaroglu et al., 2002), particularly before necrosis initiates (Tidball et al., 1995). In addition, p53 is one of the most highly induced transcription factors in *mdx* (Dogra et al., 2008), and its inhibition reduced exercise-induced necrosis in the dystrophic mouse (Waters et al., 2010), suggesting an important role in the *mdx* pathology. We identify an enrichment of “Regulation Of Apoptotic Process” genes that are differentially expressed, and a 4.2 fold increase in *ZNF385A* in TA (*p*-value = 1.76E-102), which is a reported modulator of p53 that reduces pro-apoptotic signaling (Das et al., 2007). This relatively higher expression of *ZNF385A* is also observed in gracilis (Abbassi-Daloii et al., 2023) and EOM (Porter et al., 2001; Terry et al., 2018) which are protected in DMD compared to the VL. Because of the potential impact of ZNF385A to suppress apoptosis, *ZNF385A* may protect the TA via modulation of p53 signaling towards an anti-apoptotic state. Further studies need to be conducted on 1) what are the direct or indirect targets of *ZNF385A* in human muscle, 2) whether up or downregulating the expression of *ZNF385A* has an effect on the apoptotic rate in myotubes and muscle stem cells exposed to an apoptotic-inducing condition, and 3) whether inducing its expression in dystrophic myotubes protects myofibers and other resident muscle cells from death.

DMD modifier genes were more likely to be differentially expressed between VL and TA, supporting a functional role for several modifiers from this orthogonal transcriptomic study. Identifying novel genetic modifiers of DMD remains a challenge, as studies are limited due to sample size, and thus under-powered to detect genome-wide significance. Thus, augmenting with other data types is relevant to increasing confidence in observed genetic modifiers.

The higher expression of the genetic modifier *LTBP4* in TA and its restriction to fibroblasts is consistent with a role in slowing disease progression, as it binds TGF-β and thus reduces TGF-β signaling (Flanigan et al., 2013), a major driver of fibrosis. *LTBP4* also had high expression in satellite cells, an unexpected finding. Although TGF-β signaling is known to modulate the muscle stem cell function, the specific role of *LTBP4* in satellite cells has not been elucidated. If *LTBP4* participates in regulation of satellite cell function, it may create differences in the muscle-specific regenerative ability that needs further exploration, particularly in the context of the protective IAAM haplotype.

Actinin-3 null allele has been previously reported to be protective in DMD via a shift to a more oxidative metabolism (Hogarth et al., 2017) characteristic of slow fibers, which are more protected from loss in DMD. Various studies have examined whether there is an associated change in fiber type composition in the *ACTN3* null genotype, with some finding no evidence for a fiber type shift (MacArthur et al., 2008; Broos et al., 2016), and others finding significant differences in the fiber type composition across genotype groups (Vincent et al., 2007). The discrepancies could be due to different sampling methods, such as the number of fibers counted. The higher proportion of slow fibers in XX individuals may be protective because slow fibers are protected for longer in DMD (Webster et al., 1988). We detected previously unreported expression of actinin-3 in slow fibers at low levels, particularly in the RR and RX groups. The presence of actinin-3 in slow fibers in the VL may render VL slow fibers more susceptible to damage by increasing glycolytic and reducing oxidative metabolism.

Differential mutual exclusion of exons 143 and 144 of *NEB*, as we observed here, has been observed for another pair of human muscles, and gastrocnemius (GN) preferentially includes exon 144 and TA exon 143 (Donner et al., 2004; Lam et al., 2018), the latter being consistent with this study. Similar to the difference in progression between the VL and TA in DMD where the TA is delayed by about 8.5 years, the TA is delayed by 3.4 years relative to GN (Rooney et al., 2020). Because the more affected VL and GN preferentially include E144 and not E143, we hypothesize that E143 included nebulin could plausibly confer different sarcomere properties that result in protection of the muscle membrane to contraction-induced injury in the absence of dystrophin.

Nebulin has various roles in skeletal muscle. Although the most commonly known role is thin filament length regulation and stabilization, it also has been recently found to have roles in modulating contractile force, calcium handling, and the actin-myosin interaction (Chu et al., 2016). Mutations in *NEB* are the most common cause of autosomal-recessive nemaline myopathy, characterized by Z-disk and thin filament proteins aggregated into nemaline bodies, Z-disk disorganization and consequently, early-onset muscle weakness that mainly affect proximal muscles (Lehtokari et al., 2014). Homozygous intronic mutations in intron 144, which created an alternative donor (5’) splice site in exon 144 and a decrease in *NEB* expression, were found causal in a case of a 6-year-old boy with general muscle weakness and nemaline bodies consistent with nemaline myopathy (Laflamme et al., 2021). Exons 143 and 144 encode the super repeat region 21 (S21) of nebulin (Lam et al., 2018) and how they differ functionally has not been extensively studied. The only reported difference is in their charge, hydrophobicity and the predicted presence of a protein kinase C phosphorylation site in the E144 but not in the E143 (Donner et al., 2004). The central super repeat region, which has 22 super repeats in total, has been proposed to interact with KLHL40 (Garg et al., 2014). KLHL40 is located in the sarcomere I and A bands, where it binds to nebulin (Garg et al., 2014). Similar to mutations in the *NEB* exon 143-144 region, KLHL40 deficiency is associated with nemaline myopathy (Garg et al., 2014). These data indicate that the S21 repeat region is critical for proper sarcomere organization, and consequently, muscle function. Nebulin S21 isoforms with different charge and hydrophobicity can potentially modulate the sarcomere organization, structure, and stability and lead to a different susceptibility of the dystrophin-glycoprotein-sarcomere link to damage in the absence of dystrophin.

Previous reports on isoform switching across leg muscles identified 200 switching isoforms among 79 genes (Abbassi-Daloii et al., 2023). However, we did not identify any of these isoforms switch events between VL and TA. These findings could be partially attributed to the different skeletal muscles studied, RNA quality and the sequencing library type. In our study, we utilized ribosomal depletion before cDNA synthesis. However, poly(A) libraries can be 3’ end biased (Shi et al., 2021) and this can affect isoform quantification.

This study further provides insights into transcriptomic signatures of differentially affected muscle groups, at both the gene and isoform level, and constitutes the first study, to our knowledge, to augment transcriptomic data from different healthy human skeletal muscles using single nuclei transcriptomics to unravel the complexity of tissue heterogeneity and its contribution to intrinsic transcriptomic signatures. To our knowledge, this study also generated the second and largest reported DMD bulk RNAseq dataset, from young ambulatory patients with the same type of *DMD* mutation (nonsense mutation). An existing dataset of 5 DMD muscle RNAseq (sequenced muscle not specified) can be found in the Sequence Read Archive (SRA) database (PRJNA734152), and RNAseq for four different muscles (1 biceps, 1 quadriceps, 1 gastrocnemius, 1 tibialis anterior) can be found in PRJNA342787. In addition, this is the first throughput dataset of the DMD TA. Although various other datasets of human DMD muscle microarray are found in the Gene Expression Omnibus (GEO) database (GSE3307, GSE109178, GSE6011, GSE1004, GSE38417, GSE13608), GSE6011 is the microarray dataset at the earliest stage reported, and it corresponds to the quadriceps (at times used to refer to the VL) at less than 2-year of age. Considering that our DMD TA dataset is from muscle at 2–7 years of age, and that the TA is protected for 8.5 years compared to the VL (Rooney et al., 2020), we estimate that the herein generated TA dataset is the DMD whole muscle transcriptome at the earliest stage of the disease reported to date. Furthermore, the healthy snRNAseq and bulk RNAseq datasets provide useful resources for identification of muscle disease genes through transcriptomics, which require healthy reference materials (Lee et al., 2020). In addition, the snRNAseq dataset could be used to identify splicing factors co-expressed in single cells predominantly expressing different isoforms, and various methods have been developed to overcome the challenges of isoform quantification caused by 3’ bias, low sequencing depth and dropout (Huang and Sanguinetti, 2017; Song et al., 2017; Hu et al., 2020; Pan et al., 2021). Establishing a single nuclei atlas of healthy human muscles will allow for a better understanding of muscle-specific responses to lack of dystrophin in particular cell types, how genetic modifiers may influence these, whether there is a preferential responsiveness of specific muscle groups to therapeutic approaches and what the cellular underlying mechanisms are, and how to mimic these intrinsic mechanisms to improve the effectiveness of current therapeutics.

## Data Availability

The raw RNAseq and snRNAseq data generated and analyzed in this study can be found in the Sequence Read Archive (SRA) (BioProject ID: PRJNA976807, https://www.ncbi.nlm.nih.gov/sra/PRJNA976807). The Seurat object for the snRNAseq data can be found in https://www.synapse.org/#!Synapse:syn51794252/.

## Supplementary material

The Supplementary Material for this article can be found online at: https://www.frontiersin.org/articles/10.3389/fgene.2023.1216066/full#supplementary-material. Supplementary table descriptions can be found in Data Sheet 1.

## References

[B1] Abbassi-Daloii T. el Abdellaoui S. Voortman L. M. Veeger T. T. J. Cats D. Mei H. (2023). A transcriptome atlas of leg muscles from healthy human volunteers reveals molecular and cellular signatures associated with muscle location. eLife 12, e80500. 10.7554/eLife.80500 36744868 PMC9988256

[B2] Al-Khalili Szigyarto C. (2020). Duchenne muscular dystrophy: Recent advances in protein biomarkers and the clinical application. Expert Rev. Proteomics 17 (5), 365–375. 10.1080/14789450.2020.1773806 32713262

[B3] Alonso-Jiménez A. Fernández-Simón E. Natera-de Benito D. Ortez C. García C. Montiel E. (2021). Platelet derived growth factor-AA correlates with muscle function tests and quantitative muscle magnetic resonance in dystrophinopathies. Front. Neurol. 12, 659922. 10.3389/fneur.2021.659922 34177765 PMC8226260

[B4] Anders S. Reyes A. Huber W. (2012). Detecting differential usage of exons from RNA-seq data. Genome Res. 22 (10), 2008–2017. 10.1101/gr.133744.111 22722343 PMC3460195

[B5] Barthelemy F. Woods J. D. Nieves-Rodriguez S. Douine E. D. Wang R. Wanagat J. (2020). A well-tolerated core needle muscle biopsy process suitable for children and adults. Muscle and Nerve 62 (6), 688–698. 10.1002/mus.27041 32820569 PMC7756388

[B6] Bello L. Flanigan K. M. Weiss R. B. Dunn D. M. Swoboda K. J. Gappmaier E. (2016). Association study of exon variants in the NF-κB and TGFβ pathways identifies CD40 as a modifier of duchenne muscular dystrophy. Am. J. Hum. Genet. 99 (5), 1163–1171. 10.1016/j.ajhg.2016.08.023 27745838 PMC5097949

[B7] Bencze M. (2023). Mechanisms of myofibre death in muscular dystrophies: The emergence of the regulated forms of necrosis in myology. Int. J. Mol. Sci. 24 (1), 362. 10.3390/ijms24010362 PMC9820579 36613804

[B8] Bowman A. L. Kontrogianni-Konstantopoulos A. Hirsch S. S. Geisler S. B. Gonzalez-Serratos H. Russell M. W. (2007). Different obscurin isoforms localize to distinct sites at sarcomeres. FEBS Lett. 581 (8), 1549–1554. 10.1016/j.febslet.2007.03.011 17382936 PMC1899168

[B9] Bray N. L. Pimentel H. Melsted P. Pachter L. (2016). Near-optimal probabilistic RNA-seq quantification. Nat. Biotechnol. 34 (5), 525–527. 10.1038/nbt.3519 27043002

[B10] Brinegar A. E. Xia Z. Loehr J. A. Li W. Rodney G. G. Cooper T. A. (2017). Extensive alternative splicing transitions during postnatal skeletal muscle development are required for calcium handling functions. eLife 6, e27192. 10.7554/eLife.27192 28826478 PMC5577920

[B11] Broos S. Malisoux L. Theisen D. van Thienen R. Ramaekers M. Jamart C. (2016). Evidence for ACTN3 as a speed gene in isolated human muscle fibers. PLOS ONE 11 (3), e0150594. 10.1371/journal.pone.0150594 26930663 PMC4773019

[B12] Chen E. Y. Tan C. M. Kou Y. Duan Q. Wang Z. Meirelles G. V. (2013). Enrichr: Interactive and collaborative HTML5 gene list enrichment analysis tool. BMC Bioinforma. 14 (1), 128. 10.1186/1471-2105-14-128 PMC3637064 23586463

[B13] Chen X. Li Y. (2009). Role of matrix metalloproteinases in skeletal muscle: Migration, differentiation, regeneration and fibrosis. Cell Adhesion Migr. 3 (4), 337–341. 10.4161/cam.3.4.9338 PMC2802742 19667757

[B14] Chu M. Gregorio C. C. Pappas C. T. (2016). Nebulin, a multi-functional giant. J. Exp. Biol. 219 (2), 146–152. 10.1242/jeb.126383 26792324 PMC6514474

[B15] Cohen E. Bonne G. Rivier F. Hamroun D. (2021). The 2022 version of the gene table of neuromuscular disorders (nuclear genome). Neuromuscul. Disord. 31 (12), 1313–1357. 10.1016/j.nmd.2021.11.004 34930546

[B16] Costantini A. Trevi E. Pala M. I. Fancellu R. (2016). Can long-term thiamine treatment improve the clinical outcomes of myotonic dystrophy type 1? Neural Regen. Res. 11 (9), 1487–1491. 10.4103/1673-5374.191225 27857755 PMC5090854

[B17] Das S. Raj L. Zhao B. Kimura Y. Bernstein A. Aaronson S. A. (2007). Hzf determines cell survival upon genotoxic stress by modulating p53 transactivation. Cell 130 (4), 624–637. 10.1016/j.cell.2007.06.013 17719541 PMC2779720

[B18] de Zélicourt A. Fayssoil A. Dakouane-Giudicelli M. De Jesus I. Karoui A. Zarrouki F. (2022). CD38-NADase is a new major contributor to Duchenne muscular dystrophic phenotype. EMBO Mol. Med. 14 (5), e12860. 10.15252/emmm.202012860 35298089 PMC9081905

[B19] Deconinck A. E. Rafael J. A. Skinner J. A. Brown S. C. Potter A. C. Metzinger L. (1997). Utrophin-dystrophin-deficient mice as a model for duchenne muscular dystrophy. Cell 90 (4), 717–727. 10.1016/S0092-8674(00)80532-2 9288751

[B20] Dobin A. Davis C. A. Schlesinger F. Drenkow J. Zaleski C. Jha S. (2012). Star: Ultrafast universal RNA-seq aligner. Bioinformatics 29 (1), 15–21. 10.1093/bioinformatics/bts635 23104886 PMC3530905

[B21] Dogra C. Srivastava D. S. Kumar A. (2008). Protein–DNA array-based identification of transcription factor activities differentially regulated in skeletal muscle of normal and dystrophin-deficient mdx mice. Mol. Cell. Biochem. 312 (1), 17–24. 10.1007/s11010-008-9716-6 18278580 PMC2785438

[B22] Donner K. Sandbacka M. Lehtokari V.-L. Wallgren-Pettersson C. Pelin K. (2004). Complete genomic structure of the human nebulin gene and identification of alternatively spliced transcripts. Eur. J. Hum. Genet. 12 (9), 744–751. 10.1038/sj.ejhg.5201242 15266303

[B23] Edgerton V. R. Smith J. L. Simpson D. R. (1975). Muscle fibre type populations of human leg muscles. Histochem. J. 7 (3), 259–266. 10.1007/BF01003594 123895

[B24] Fanin M. Danieli G. A. Cadaldini M. Miorin M. Vitiello L. Angelini C. (1995). Dystrophin-positive fibers in duchenne dystrophy: Origin and correlation to clinical course. Muscle and Nerve 18 (10), 1115–1120. 10.1002/mus.880181007 7659105

[B25] Fischer M. D. Gorospe J. R. Felder E. Bogdanovich S. Pedrosa-Domellöf F. Ahima R. S. (2002). Expression profiling reveals metabolic and structural components of extraocular muscles. Physiol. Genomics 9 (2), 71–84. 10.1152/physiolgenomics.00115.2001 12006673

[B26] Flanigan K. M. Ceco E. Lamar K.-M. Kaminoh Y. Dunn D. M. Mendell J. R. (2013). LTBP4 genotype predicts age of ambulatory loss in duchenne muscular dystrophy. Ann. Neurology 73 (4), 481–488. 10.1002/ana.23819 PMC4106425 23440719

[B27] Flanigan K. M. Waldrop M. A. Martin P. T. Alles R. Dunn D. M. Alfano L. N. (2023). A genome-wide association analysis of loss of ambulation in dystrophinopathy patients suggests multiple candidate modifiers of disease severity. Eur. J. Hum. Genet. 2023, 663–673. 10.1038/s41431-023-01329-5 PMC10250491 36935420

[B28] Garg A. O'Rourke J. Long C. Doering J. Ravenscroft G. Bezprozvannaya S. (2014). KLHL40 deficiency destabilizes thin filament proteins and promotes nemaline myopathy. J. Clin. Invest. 124 (8), 3529–3539. 10.1172/jci74994 24960163 PMC4109545

[B29] Gholamalizadeh M. Jarrahi A. M. Akbari M. E. Rezaei S. Doaei S. Mokhtari Z. (2019). The possible mechanisms of the effects of IRX3 gene on body weight: An overview. Archives Med. Sci. – Atheroscler. Dis. 4 (1), 225–230. 10.5114/amsad.2019.87545 PMC6749179 31538128

[B30] Giulietti M. Piva F. D'Antonio M. D'Onorio De Meo P. Paoletti D. Castrignanò T. (2013). SpliceAid-F: A database of human splicing factors and their RNA-binding sites. Nucleic Acids Res. 41, D125–D131. Database issue). 10.1093/nar/gks997 23118479 PMC3531144

[B31] Grounds M. D. Terrill J. R. Al-Mshhdani B. A. Duong M. N. Radley-Crabb H. G. Arthur P. G. (2020). Biomarkers for duchenne muscular dystrophy: Myonecrosis, inflammation and oxidative stress. Dis. Model Mech. 13 (2), dmm043638. 10.1242/dmm.043638 32224496 PMC7063669

[B32] Hämäläinen N. Pette D. (1993). The histochemical profiles of fast fiber types IIB, IID, and IIA in skeletal muscles of mouse, rat, and rabbit. J. Histochem Cytochem 41 (5), 733–743. 10.1177/41.5.8468455 8468455

[B33] Han R. Rader E. P. Levy J. R. Bansal D. Campbell K. P. (2011). Dystrophin deficiency exacerbates skeletal muscle pathology in dysferlin-null mice. Skelet. Muscle 1 (1), 35. 10.1186/2044-5040-1-35 22132688 PMC3287108

[B34] Hao Y. Hao S. Andersen-Nissen E. Mauck W. M. Zheng S. Butler A. (2021). Integrated analysis of multimodal single-cell data. Cell 184 (13), 3573–3587.e29. 10.1016/j.cell.2021.04.048 34062119 PMC8238499

[B35] Hathout Y. Marathi R. L. Rayavarapu S. Zhang A. Brown K. J. Seol H. (2014). Discovery of serum protein biomarkers in the mdx mouse model and cross-species comparison to Duchenne muscular dystrophy patients. Hum. Mol. Genet. 23 (24), 6458–6469. 10.1093/hmg/ddu366 25027324 PMC4240201

[B36] Hirokawa N. Noda Y. (2008). Intracellular transport and kinesin superfamily proteins, KIFs: Structure, function, and dynamics. Physiol. Rev. 88 (3), 1089–1118. 10.1152/physrev.00023.2007 18626067

[B37] Hoffman E. P. Brown R. H. Jr. Kunkel L. M. (1987). Dystrophin: The protein product of the duchenne muscular dystrophy locus. Cell 51 (6), 919–928. 10.1016/0092-8674(87)90579-4 3319190

[B38] Hogarth M. W. Houweling P. J. Thomas K. C. Gordish-Dressman H. Bello L. Vishwanathan V. (2017). Evidence for ACTN3 as a genetic modifier of Duchenne muscular dystrophy. Nat. Commun. 8 (1), 14143. 10.1038/ncomms14143 28139640 PMC5290331

[B39] Hu Y. Wang K. Li M. (2020). Detecting differential alternative splicing events in scRNA-seq with or without Unique Molecular Identifiers. PLoS Comput. Biol. 16 (6), e1007925. 10.1371/journal.pcbi.1007925 32502143 PMC7299405

[B40] Huang Y. Sanguinetti G. (2017). Brie: Transcriptome-wide splicing quantification in single cells. Genome Biol. 18 (1), 123. 10.1186/s13059-017-1248-5 28655331 PMC5488362

[B41] Jakobsson F. Edström L. Grimby L. Thornell L. E. (1991). Disuse of anterior tibial muscle during locomotion and increased proportion of type II fibres in hemiplegia. J. Neurological Sci. 105 (1), 49–56. 10.1016/0022-510X(91)90117-P 1795169

[B42] Joe M. K. Kee C. Tomarev S. I. (2012). Myocilin interacts with syntrophins and is member of dystrophin-associated protein complex. J. Biol. Chem. 287 (16), 13216–13227. 10.1074/jbc.M111.224063 22371502 PMC3339941

[B43] Judge S. M. Deyhle M. R. Neyroud D. Nosacka R. L. D'Lugos A. C. Cameron M. E. (2020). MEF2c-Dependent downregulation of myocilin mediates cancer-induced muscle wasting and associates with cachexia in patients with cancer. Cancer Res. 80 (9), 1861–1874. 10.1158/0008-5472.Can-19-1558 32132110 PMC7250164

[B44] Kaminski H. J. Al-Hakim M. Leigh R. J. Bashar M. K. Ruff R. L. (1992). Extraocular muscles are spared in advanced duchenne dystrophy. Ann. Neurology 32 (4), 586–588. 10.1002/ana.410320418 1456746

[B45] Karpati G. Carpenter S. Prescott S. (1988). Small-caliber skeletal muscle fibers do not suffer necrosis in mdx mouse dystrophy. Muscle and Nerve 11 (8), 795–803. 10.1002/mus.880110802 3173406

[B46] Kute P. M. Soukarieh O. Tjeldnes H. Trégouët D.-A. Valen E. (2022). Small open reading frames, how to find them and determine their function. Front. Genet. 12, 796060. 10.3389/fgene.2021.796060 35154250 PMC8831751

[B47] Laflamme N. Lace B. Thonta Setty S. Rioux N. Labrie Y. Droit A. (2021). A homozygous deep intronic mutation alters the splicing of nebulin gene in a patient with nemaline myopathy. Front. Neurol. 12, 660113. 10.3389/fneur.2021.660113 34211429 PMC8239344

[B48] Lam L. T. Holt I. Laitila J. Hanif M. Pelin K. Wallgren-Pettersson C. (2018). Two alternatively-spliced human nebulin isoforms with either exon 143 or exon 144 and their developmental regulation. Sci. Rep. 8 (1), 15728. 10.1038/s41598-018-33281-6 30356055 PMC6200726

[B49] Lee H. Huang A. Y. Wang L.-k. Yoon A. J. Renteria G. Eskin A. (2020). Diagnostic utility of transcriptome sequencing for rare Mendelian diseases. Genet. Med. 22 (3), 490–499. 10.1038/s41436-019-0672-1 31607746 PMC7405636

[B50] Lee-Gannon T. Jiang X. Tassin T. C. Mammen P. P. A. (2022). Biomarkers in duchenne muscular dystrophy. Curr. Heart Fail. Rep. 19 (2), 52–62. 10.1007/s11897-022-00541-6 35386072

[B51] Lehtokari V. L. Kiiski K. Sandaradura S. A. Laporte J. Repo P. Frey J. A. (2014). Mutation update: The spectra of nebulin variants and associated myopathies. Hum. Mutat. 35 (12), 1418–1426. 10.1002/humu.22693 25205138 PMC4295925

[B52] Li H. Xiao L. Wang L. Lin J. Luo M. Chen M. (2018). HLA Polymorphism Affects Risk of de novo Mutation of dystrophin Gene and Clinical Severity of Duchenne Muscular Dystrophy in a Southern Chinese Population. Front. Neurology 9, 970. 10.3389/fneur.2018.00970 PMC6249334 30498470

[B53] Loreti M. Sacco A. (2022). The jam session between muscle stem cells and the extracellular matrix in the tissue microenvironment. npj Regen. Med. 7 (1), 16. 10.1038/s41536-022-00204-z 35177651 PMC8854427

[B54] Love M. I. Huber W. Anders S. (2014). Moderated estimation of fold change and dispersion for RNA-seq data with DESeq2. Genome Biol. 15 (12), 550. 10.1186/s13059-014-0550-8 25516281 PMC4302049

[B55] MacArthur D. G. Seto J. T. Chan S. Quinlan K. G. R. Raftery J. M. Turner N. (2008). An Actn3 knockout mouse provides mechanistic insights into the association between alpha-actinin-3 deficiency and human athletic performance. Hum. Mol. Genet. 17 (8), 1076–1086. 10.1093/hmg/ddm380 18178581

[B56] Mann C. J. Perdiguero E. Kharraz Y. Aguilar S. Pessina P. Serrano A. L. (2011). Aberrant repair and fibrosis development in skeletal muscle. Skelet. Muscle 1 (1), 21. 10.1186/2044-5040-1-21 21798099 PMC3156644

[B57] Matsakas A. Yadav V. Lorca S. Narkar V. (2013). Muscle ERRγ mitigates Duchenne muscular dystrophy via metabolic and angiogenic reprogramming. FASEB J. 27 (10), 4004–4016. 10.1096/fj.13-228296 23781095

[B58] McGinnis C. S. Murrow L. M. Gartner Z. J. (2019). DoubletFinder: Doublet detection in single-cell RNA sequencing data using artificial nearest neighbors. Cell Syst. 8 (4), 329–337.e4. 10.1016/j.cels.2019.03.003 30954475 PMC6853612

[B59] [DATASET] McKellar D. W. Walter L. D. Song L. T. Mantri M. Wang M. F. Z. De Vlaminck I. (2021). Large-scale integration of single-cell transcriptomic data captures transitional progenitor states in mouse skeletal muscle regeneration (scMuscle). Dryad. v1.1. 10.5061/dryad.t4b8gtj34 PMC8589952 34773081

[B60] Morales M. G. Gutierrez J. Cabello-Verrugio C. Cabrera D. Lipson K. E. Goldschmeding R. (2013). Reducing CTGF/CCN2 slows down mdx muscle dystrophy and improves cell therapy. Hum. Mol. Genet. 22 (24), 4938–4951. 10.1093/hmg/ddt352 23904456

[B61] Nakka K. Ghigna C. Gabellini D. Dilworth F. J. (2018). Diversification of the muscle proteome through alternative splicing. Skelet. Muscle 8 (1), 8. 10.1186/s13395-018-0152-3 29510724 PMC5840707

[B62] Newman A. M. Steen C. B. Liu C. L. Gentles A. J. Chaudhuri A. A. Scherer F. (2019). Determining cell type abundance and expression from bulk tissues with digital cytometry. Nat. Biotechnol. 37 (7), 773–782. 10.1038/s41587-019-0114-2 31061481 PMC6610714

[B63] Nieves Rodríguez S. (2023). Transcriptomic analysis of healthy skeletal muscle to identify modulators of differential skeletal muscle susceptibility in Duchenne muscular dystrophy. Los Angeles: Ph.D., University of California.

[B64] Pan L. Dinh H. Q. Pawitan Y. Vu T. N. (2021). Isoform-level quantification for single-cell RNA sequencing. Bioinformatics 38 (5), 1287–1294. 10.1093/bioinformatics/btab807 PMC8826380 34864849

[B65] Park S. Ranjbarvaziri S. Zhao P. Ardehali R. (2020). Cardiac fibrosis is associated with decreased circulating levels of full-length CILP in heart failure. JACC Basic Transl. Sci. 5 (5), 432–443. 10.1016/j.jacbts.2020.01.016 32478206 PMC7251193

[B66] Parolo S. Marchetti L. Lauria M. Misselbeck K. Scott-Boyer M. P. Caberlotto L. (2018). Combined use of protein biomarkers and network analysis unveils deregulated regulatory circuits in Duchenne muscular dystrophy. PLoS One 13 (3), e0194225. 10.1371/journal.pone.0194225 29529088 PMC5846794

[B67] Pegoraro E. Hoffman E. P. Piva L. Gavassini B. F. Cagnin S. Ermani M. (2011). SPP1 genotype is a determinant of disease severity in Duchenne muscular dystrophy. Neurology 76 (3), 219–226. 10.1212/WNL.0b013e318207afeb 21178099 PMC3034396

[B68] [DATASET] Petrany M. J. Swoboda C. O. Sun C. Chetal K. Chen X. Weirauch M. T. (2020). snRNA-Seq of multinucleated skeletal myofibers (myoatlas), Synapse. v1. syn21676145 (syn51119242). 10.1038/s41467-020-20063-w PMC7733460 33311464

[B69] Petrof B. J. Shrager J. B. Stedman H. H. Kelly A. M. Sweeney H. L. (1993). Dystrophin protects the sarcolemma from stresses developed during muscle contraction. Proc. Natl. Acad. Sci. U. S. A. 90 (8), 3710–3714. 10.1073/pnas.90.8.3710 8475120 PMC46371

[B70] Pettygrove S. Lu Z. Andrews J. G. Meaney F. J. Sheehan D. W. Price E. T. (2014). Sibling concordance for clinical features of Duchenne and Becker muscular dystrophies. Muscle and Nerve 49 (6), 814–821. 10.1002/mus.24078 24030636 PMC4481732

[B71] Porter J. D. (2002). Extraocular muscle: Cellular adaptations for a diverse functional repertoire. Ann. N. Y. Acad. Sci. 956 (1), 7–16. 10.1111/j.1749-6632.2002.tb02804.x 11960789

[B72] Porter J. D. Khanna S. Kaminski H. J. Rao J. S. Merriam A. P. Richmonds C. R. (2001). Extraocular muscle is defined by a fundamentally distinct gene expression profile. Proc. Natl. Acad. Sci. U. S. A. 98 (21), 12062–12067. 10.1073/pnas.211257298 11572940 PMC59827

[B73] Resch Z. T. Fautsch M. P. (2009). Glaucoma-associated myocilin: A better understanding but much more to learn. Exp. Eye Res. 88 (4), 704–712. 10.1016/j.exer.2008.08.011 18804106 PMC2682697

[B74] Ritchie M. E. Phipson B. Wu D. Hu Y. Law C. W. Shi W. (2015). Limma powers differential expression analyses for RNA-sequencing and microarray studies. Nucleic Acids Res. 43 (7), e47. 10.1093/nar/gkv007 25605792 PMC4402510

[B75] Rooney W. D. Berlow Y. A. Triplett W. T. Forbes S. C. Willcocks R. J. Wang D. J. (2020). Modeling disease trajectory in Duchenne muscular dystrophy. Neurology 94 (15), e1622–e1633. 10.1212/wnl.0000000000009244 32184340 PMC7251517

[B76] Sandri M. Minetti C. Pedemonte M. Carraro U. (1998). Apoptotic myonuclei in human Duchenne muscular dystrophy. Lab. Invest. 78 (8), 1005–1016. 9714187

[B77] Savarese M. Jonson P. H. Huovinen S. Paulin L. Auvinen P. Udd B. (2018). The complexity of titin splicing pattern in human adult skeletal muscles. Skelet. Muscle 8 (1), 11. 10.1186/s13395-018-0156-z 29598826 PMC5874998

[B78] Schneider C. A. Rasband W. S. Eliceiri K. W. (2012). NIH image to ImageJ: 25 years of image analysis. Nat. Methods 9 (7), 671–675. 10.1038/nmeth.2089 22930834 PMC5554542

[B79] Scripture-Adams D. D. Chesmore K. N. Barthélémy F. Wang R. T. Nieves-Rodriguez S. Wang D. W. (2022). Single nuclei transcriptomics of muscle reveals intra-muscular cell dynamics linked to dystrophin loss and rescue. Commun. Biol. 5 (1), 989. 10.1038/s42003-022-03938-0 36123393 PMC9485160

[B80] Serdaroglu A. Gücüyener K. Erdem S. Köse G. Tan E. Okuyaz Ç. (2002). Role of apoptosis in duchenne's muscular dystrophy. J. Child Neurology 17 (1), 66–68. 10.1177/088307380201700120 11913578

[B81] Shi H. Zhou Y. Jia E. Pan M. Bai Y. Ge Q. (2021). Bias in RNA-seq library preparation: Current challenges and solutions. Biomed. Res. Int. 2021, 6647597. 10.1155/2021/6647597 33987443 PMC8079181

[B82] Sinha U. Malis V. Chen J. S. Csapo R. Kinugasa R. Narici M. V. (2020). Role of the extracellular matrix in loss of muscle force with age and unloading using magnetic resonance imaging, biochemical analysis, and computational models. Front. Physiol. 11, 626. 10.3389/fphys.2020.00626 32625114 PMC7315044

[B83] Song Y. Botvinnik O. B. Lovci M. T. Kakaradov B. Liu P. Xu J. L. (2017). Single-cell alternative splicing analysis with expedition reveals splicing dynamics during neuron differentiation. Mol. Cell 67 (1), 148–161.e5. 10.1016/j.molcel.2017.06.003 28673540 PMC5540791

[B84] Spitali P. Hettne K. Tsonaka R. Charrout M. van den Bergen J. Koeks Z. (2018). Tracking disease progression non-invasively in Duchenne and Becker muscular dystrophies. J. Cachexia Sarcopenia Muscle 9 (4), 715–726. 10.1002/jcsm.12304 29682908 PMC6104105

[B85] Spitali P. Zaharieva I. Bohringer S. Hiller M. Chaouch A. Roos A. (2020). TCTEX1D1 is a genetic modifier of disease progression in Duchenne muscular dystrophy. Eur. J. Hum. Genet. 28 (6), 815–825. 10.1038/s41431-019-0563-6 31896777 PMC7253478

[B86] Supek F. Bošnjak M. Škunca N. Šmuc T. (2011). REVIGO summarizes and visualizes long lists of gene ontology terms. PLOS ONE 6 (7), e21800. 10.1371/journal.pone.0021800 21789182 PMC3138752

[B87] Taglietti V. Kefi K. Rivera L. Bergiers O. Cardone N. Coulpier F. (2023). Thyroid-stimulating hormone receptor signaling restores skeletal muscle stem cell regeneration in rats with muscular dystrophy. Sci. Transl. Med. 15 (685), 5275. 10.1126/scitranslmed.add5275 36857434

[B88] Terry E. E. Zhang X. Hoffmann C. Hughes L. D. Lewis S. A. Li J. (2018). Transcriptional profiling reveals extraordinary diversity among skeletal muscle tissues. eLife 7, e34613. 10.7554/eLife.34613 29809149 PMC6008051

[B89] Tews D. S. Goebel H. H. (1997). DNA-fragmentation and expression of apoptosis-related proteins in muscular dystrophies. Neuropathology Appl. Neurobiol. 23 (4), 331–338. 10.1111/j.1365-2990.1997.tb01304.x 9292873

[B90] Tidball J. G. Albrecht D. E. Lokensgard B. E. Spencer M. J. (1995). Apoptosis precedes necrosis of dystrophin-deficient muscle. J. Cell Sci. 108 (6), 2197–2204. 10.1242/jcs.108.6.2197 7673339

[B91] Uhlén M. Fagerberg L. Hallström B. M. Lindskog C. Oksvold P. Mardinoglu A. (2015). Proteomics. Tissue-based map of the human proteome. Science 347 (6220), 1260419. 10.1126/science.1260419 25613900

[B92] Valentine B. A. Cooper B. J. Cummings J. F. de Lahunta A. (1990). Canine X-linked muscular dystrophy: Morphologic lesions. J. Neurological Sci. 97 (1), 1–23. 10.1016/0022-510X(90)90095-5 2370557

[B93] Vincent B. Bock K. D. Ramaekers M. Eede E. V. d. Leemputte M. V. Hespel P. (2007). ACTN3 (R577X) genotype is associated with fiber type distribution. Physiol. Genomics 32 (1), 58–63. 10.1152/physiolgenomics.00173.2007 17848603

[B94] Vitting-Seerup K. Sandelin A. (2017). The landscape of isoform switches in human cancers. Mol. Cancer Res. 15 (9), 1206–1220. 10.1158/1541-7786.Mcr-16-0459 28584021

[B95] Wagner K. R. Guglieri M. Ramaiah S. K. Charnas L. Marraffino S. Binks M. (2021). Safety and disease monitoring biomarkers in duchenne muscular dystrophy: Results from a phase II trial. Biomarkers Med. 15 (15), 1389–1396. 10.2217/bmm-2021-0222 34533053

[B96] Wagner K. R. McPherron A. C. Winik N. Lee S.-J. (2002). Loss of myostatin attenuates severity of muscular dystrophy in mdx mice. Ann. Neurology 52 (6), 832–836. 10.1002/ana.10385 12447939

[B97] Waters F. J. Shavlakadze T. McIldowie M. J. Piggott M. J. Grounds M. D. (2010). Use of pifithrin to inhibit p53-mediated signalling of TNF in dystrophic muscles of mdx mice. Mol. Cell. Biochem. 337 (1), 119–131. 10.1007/s11010-009-0291-2 19859789

[B98] Way M. Pope B. Cross R. A. Kendrick-Jones J. Weeds A. G. (1992). Expression of the N-terminal domain of dystrophin in *E. coli* and demonstration of binding to F-actin. FEBS Lett. 301 (3), 243–245. 10.1016/0014-5793(92)80249-G 1577159

[B99] Webster C. Silberstein L. Hays A. P. Blau H. M. (1988). Fast muscle fibers are preferentially affected in Duchenne muscular dystrophy. Cell 52 (4), 503–513. 10.1016/0092-8674(88)90463-1 3342447

[B100] Weiss R. B. Vieland V. J. Dunn D. M. Kaminoh Y. Flanigan K. M. Project f. t. U. D. (2018). Long-range genomic regulators of THBS1 and LTBP4 modify disease severity in duchenne muscular dystrophy. Ann. Neurology 84 (2), 234–245. 10.1002/ana.25283 PMC6168392 30014611

[B101] Wishart D. S. Feunang Y. D. Guo A. C. Lo E. J. Marcu A. Grant J. R. (2018). DrugBank 5.0: A major update to the DrugBank database for 2018. Nucleic Acids Res. 46 (D1), D1074–d1082. 10.1093/nar/gkx1037 29126136 PMC5753335

[B102] Woodman K. G. Coles C. A. Toulson S. L. Gibbs E. M. Knight M. McDonagh M. (2018). Benfotiamine reduces pathology and improves muscle function in <em>mdx</em> mice. bioRxiv 288621. 10.1101/288621

[B103] Wu X. Dong N. Yu L. Liu M. Jiang J. Tang T. (2022). Identification of immune-related features involved in duchenne muscular dystrophy: A bidirectional transcriptome and proteome-driven analysis. Front. Immunol. 13, 1017423. 10.3389/fimmu.2022.1017423 36483550 PMC9724784

[B104] Yamada K. Andrews C. Chan W. M. McKeown C. A. Magli A. de Berardinis T. (2003). Heterozygous mutations of the kinesin KIF21A in congenital fibrosis of the extraocular muscles type 1 (CFEOM1). Nat. Genet. 35 (4), 318–321. 10.1038/ng1261 14595441

[B105] Zhang C. L. Zhao Q. Liang H. Qiao X. Wang J. Y. Wu D. (2018). Cartilage intermediate layer protein-1 alleviates pressure overload-induced cardiac fibrosis via interfering TGF-β1 signaling. J. Mol. Cell Cardiol. 116, 135–144. 10.1016/j.yjmcc.2018.02.006 29438665

[B106] Zhang Y. Li S. Wen X. Tong H. Li S. Yan Y. (2021). MYOC promotes the differentiation of C2C12 cells by regulation of the TGF-β signaling pathways via CAV1. Biol. (Basel) 10 (7), 686. 10.3390/biology10070686 PMC8301362 34356541

[B107] Zhang Y. Parmigiani G. Johnson W. E. (2020). ComBat-seq: Batch effect adjustment for RNA-seq count data. NAR Genomics Bioinforma. 2 (3), lqaa078. 10.1093/nargab/lqaa078 PMC7518324 33015620

