# Supplementary figures and images for: Transcriptomic analysis of paired healthy human skeletal muscles to identify modulators of disease severity in DMD
Source: Front Genet. 2023 Jul 27;14:1216066. doi: 10.3389/fgene.2023.1216066 (PMC10415210; doi:10.3389/fgene.2023.1216066)

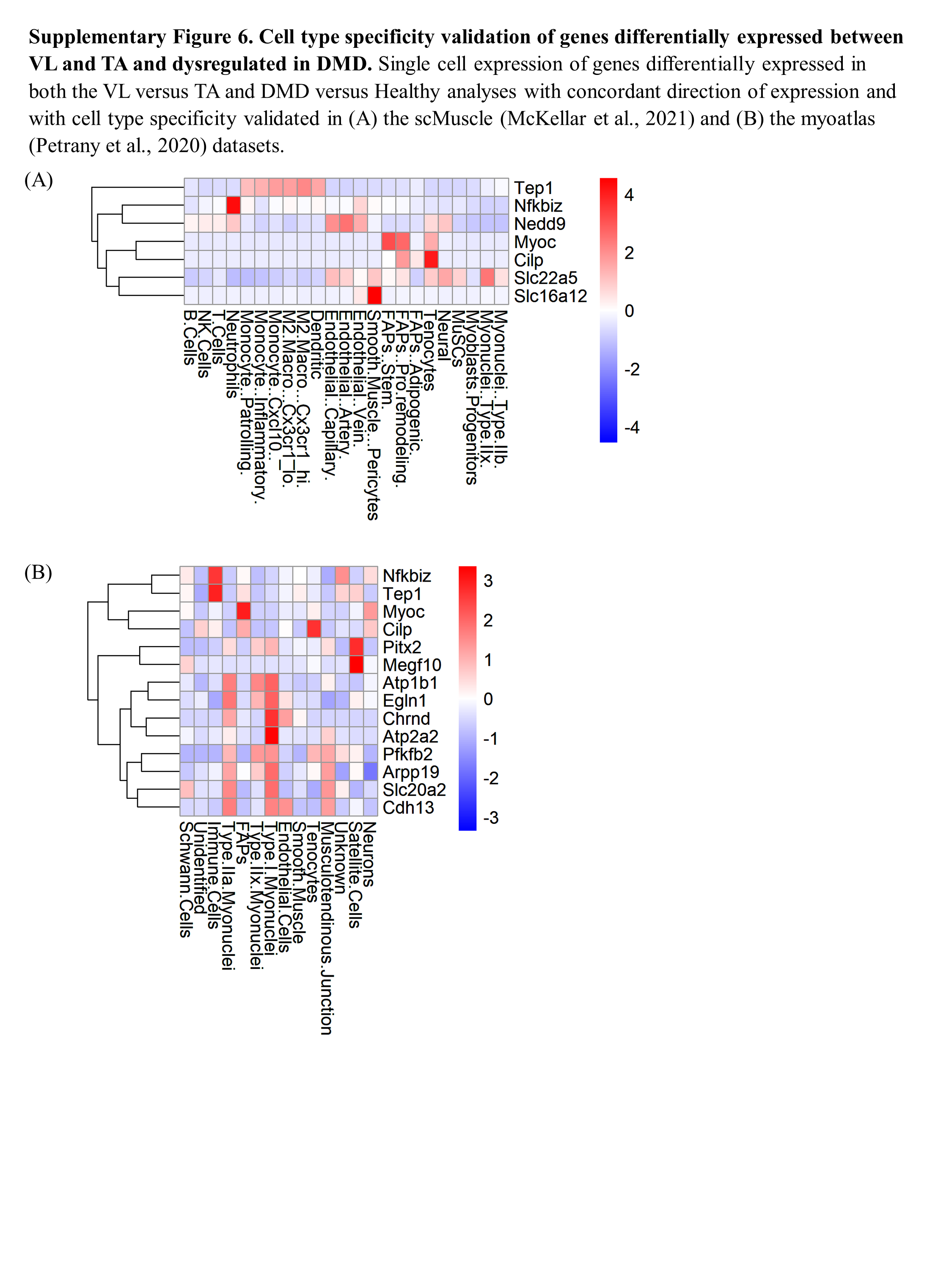

Supplement: Supplementary file 2 [file Image6.tif]

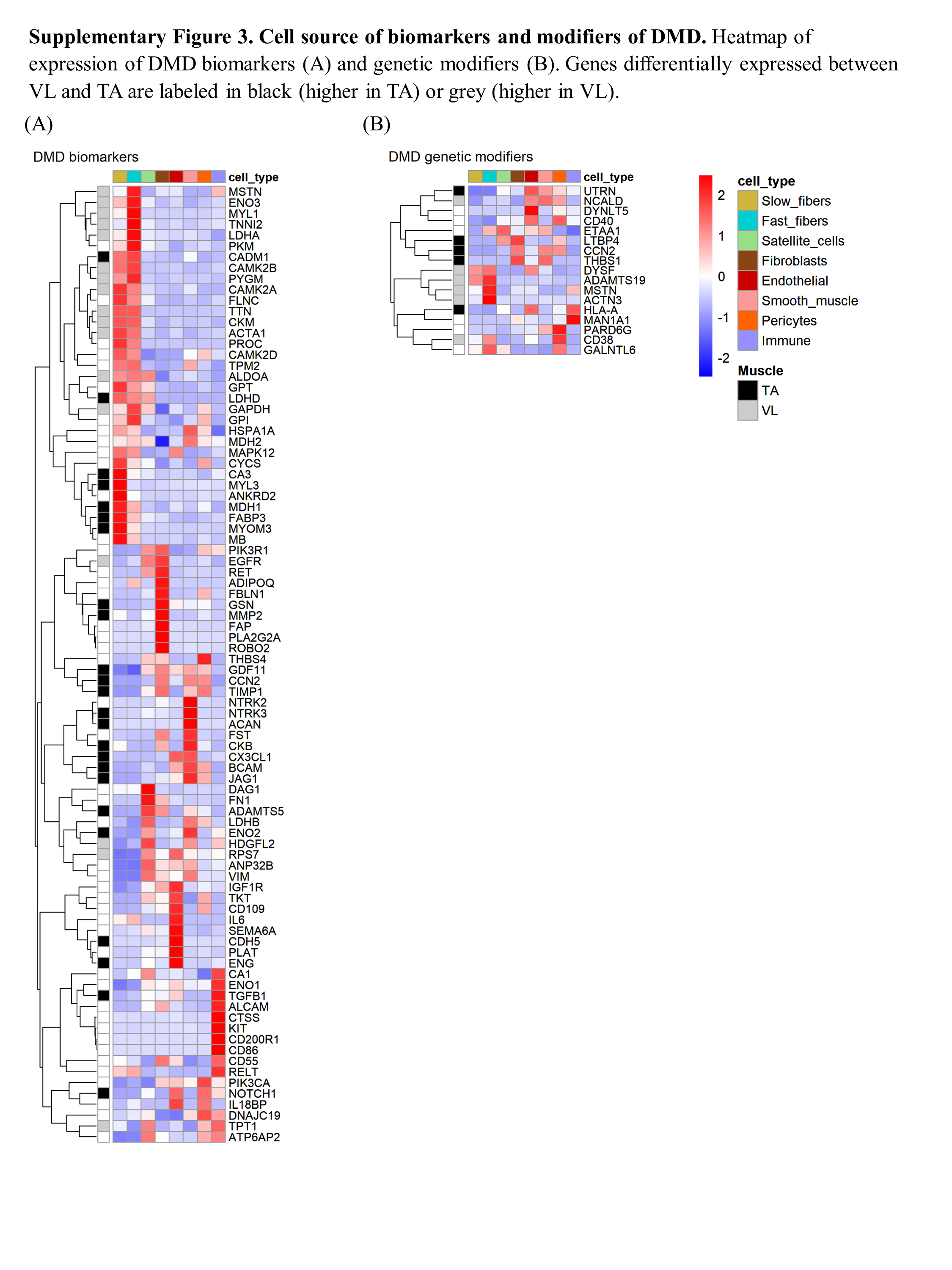

Supplement: Supplementary file 3 [file Image3.tif]

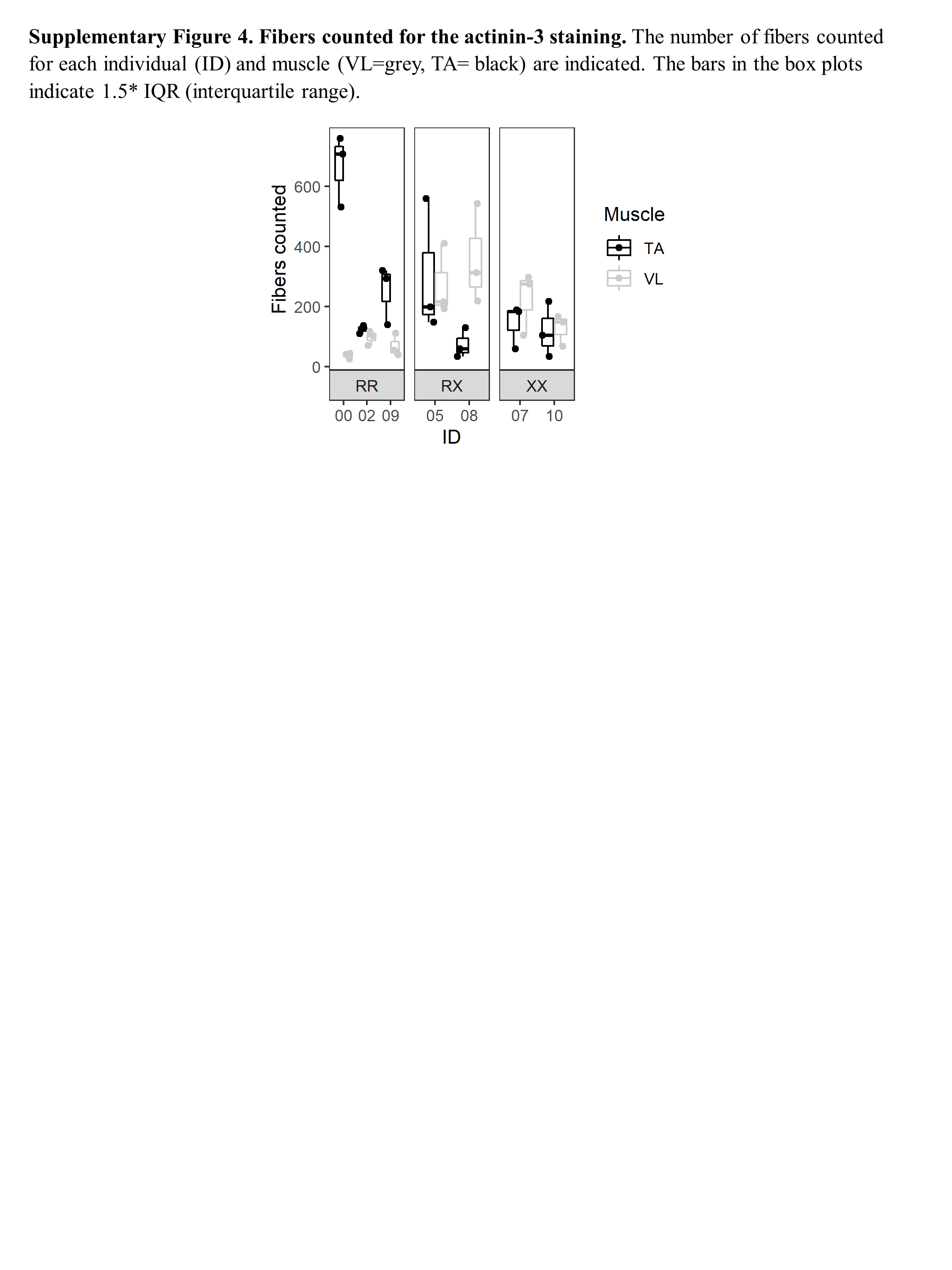

Supplement: Supplementary file 5 [file Image4.tif]

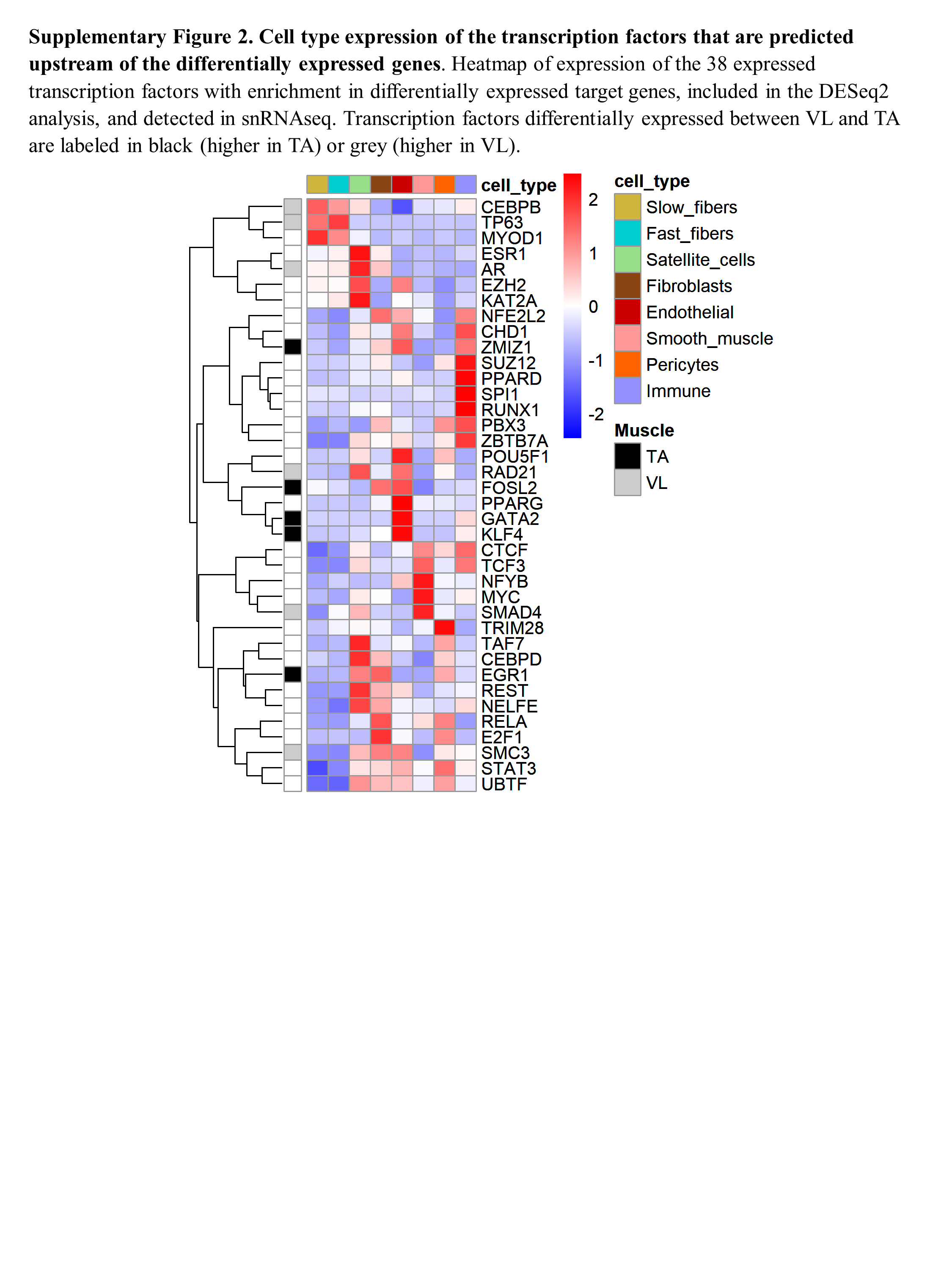

Supplement: Supplementary file 6 [file Image2.tif]

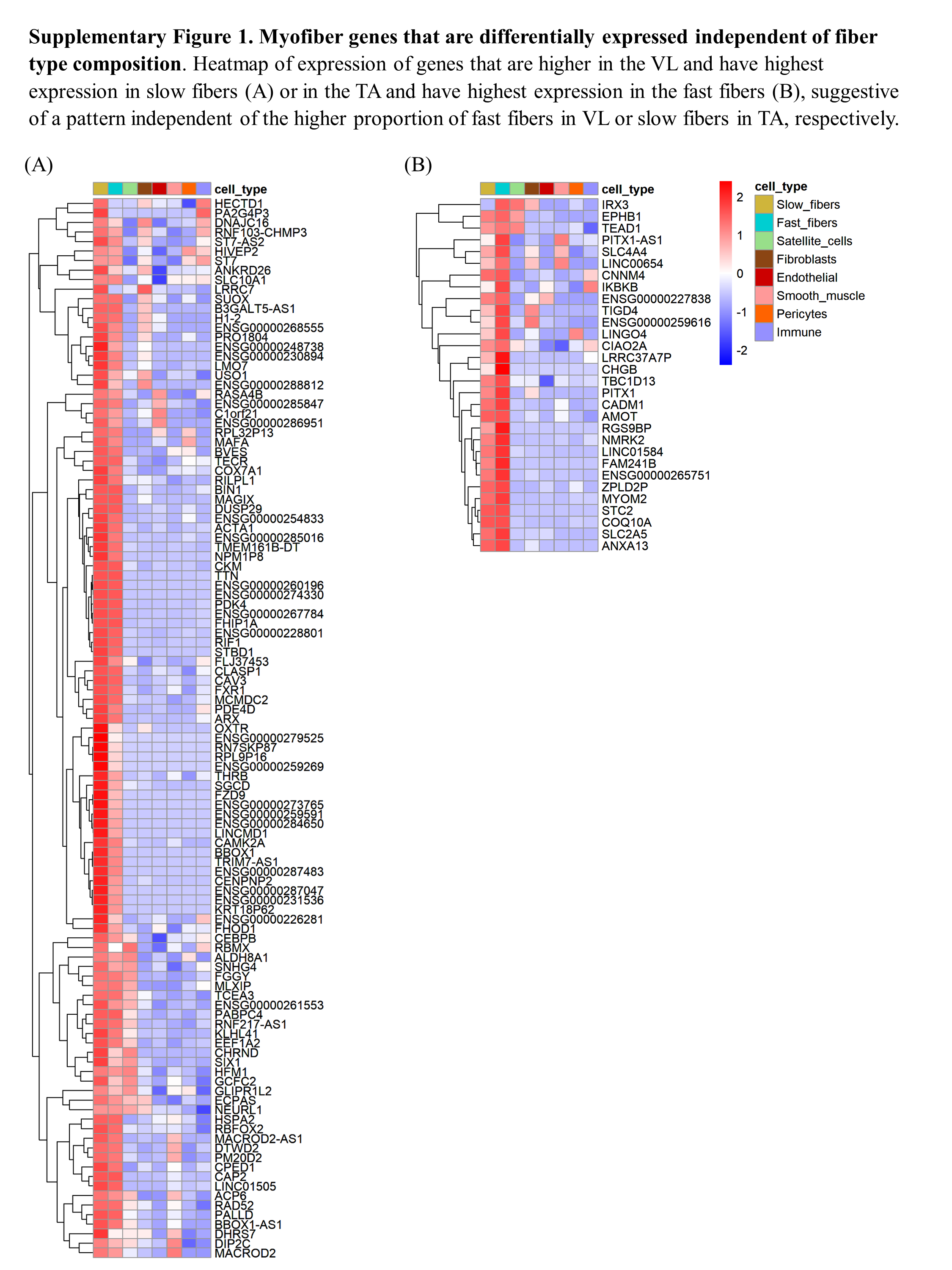

Supplement: Supplementary file 7 [file Image1.tif]

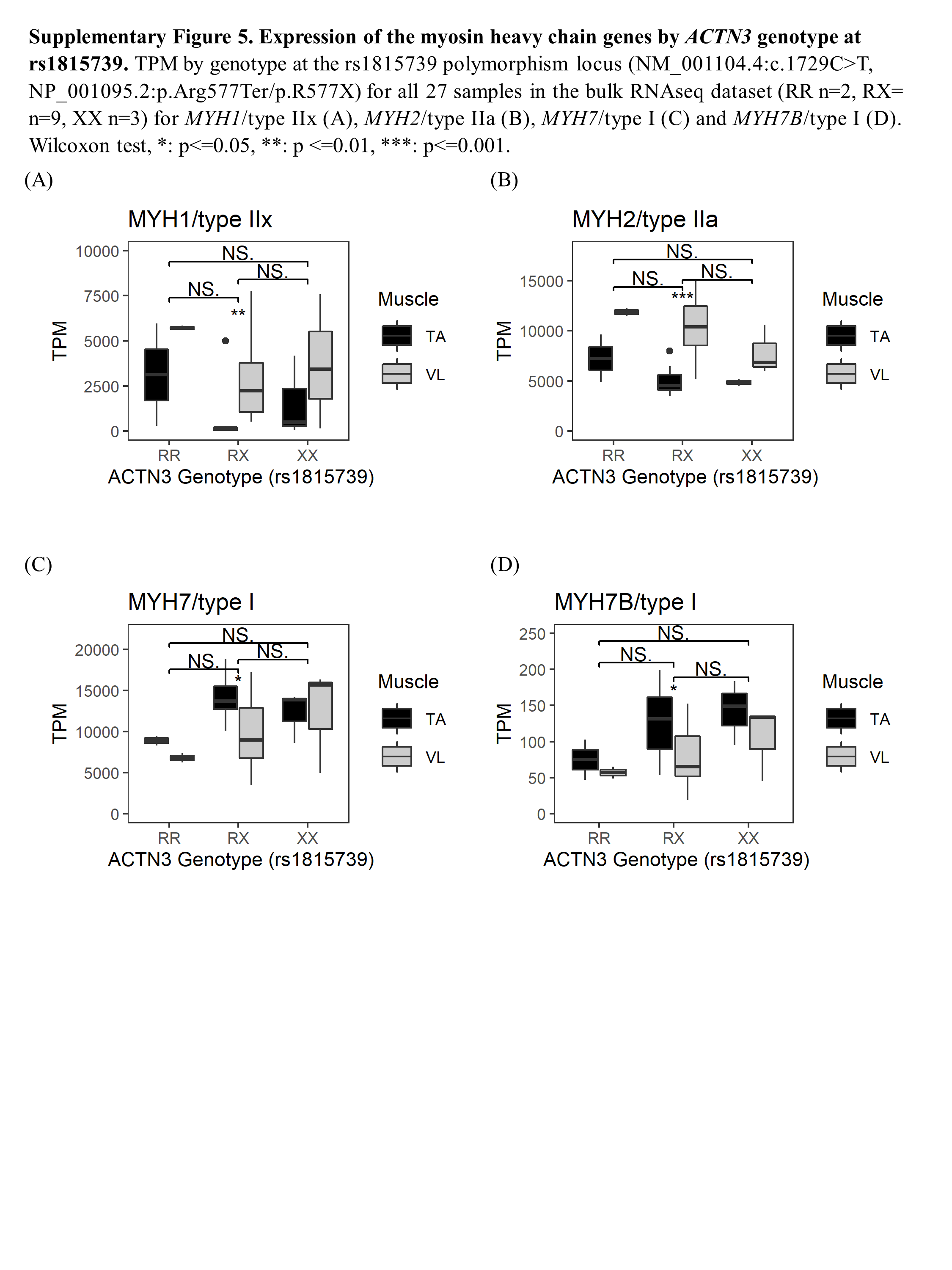

Supplement: Supplementary file 9 [file Image5.tif]
