# Supplementary material for: Transcriptomic analysis of paired healthy human skeletal muscles to identify modulators of disease severity in DMD
Source: Front Genet. 2023 Jul 27;14:1216066. doi: 10.3389/fgene.2023.1216066 (PMC10415210; doi:10.3389/fgene.2023.1216066)
Supplement: Supplementary file 10 [file DataSheet1.docx]

**Description of Supplementary Tables**

A description of the supplementary tables is included below:

**Supplementary Table S1.** DESeq2 differential gene expression analysis of VL versus TA.

-Gene type = Ensembl automatic biotype annotation for genes

-Transcription factor family = For transcription factor genes in AnimalTFDB v3.0, the family is indicated. NA = not in the database.

-Splicing Factor = For splicing factors in SpliceAid-F, the gene symbol is indicated

-baseMean = average counts

-Median TPM = median TPM across all samples

-pvalue adjusted = DESeq2 adjusted p-value. NA = not included in the DESeq2 analysis.

-Drugbank Drug ID and DrugBank Drug Common name = DrugBank drug ID and common name is included only for the differentially expressed genes.

-MaxAvgExp = Cell type where the gene has the maximum average expression (from the human VL/TA snRNAseq data).

-Columns O-V = average expression (TP10K) of genes in the snRNAseq data. NA = not detected in snRNAseq.

**Supplementary Table S2A:** EnrichR significant GO terms for Biological Process (GO_BP) and Cellular Component (GO_CC) for all 3410 differentially expressed genes and the 22414 expressed genes as background set.

-Size = Number of differentially expressed genes in the GO term

-TotalGO = Total number of genes in the GO term

-Combined.Score = P-value * Z-score

-Genes = Differentially expressed genes in the GO term category

**Supplementary Table S2B:** The most representative GO terms from ReviGO (103 total) are shown sorted by ascending p-value.

-Columns K-L: Uniqueness and Dispensability from the ReviGO summarization

-Uniqueness = Measurement of whether the term is an outlier compared to the other terms in the list. 1-(average similarity)

-Dispensability = Similarity threshold that removed a term from the list. Representative GO terms have a dispensability less than the similarity cutoff specified in the parameters.

**Supplementary Table S2C:** Positive gene markers for the 8 main cell populations obtained with Seurat’s FindAllMarkers() function (only.pos=TRUE, min.pct=0.25, logfc.threshold=0.25, random.seed =1, slot = “counts”)

-avg_log2FC = average log2(fold change)

-pct.1 = percentage of cells in the cluster where the gene is detected

-pct.2 = percentage of cells in the remaining clusters where the gene is detected

-p_val_adj = adjusted p-value

**Supplementary Table S2D:** EnrichR significant transcription factors (Term) from ENCODE_and_ChEA_Consensus_TFs_from_ChIP-X for all 3410 differentially expressed genes and the 22414 expressed genes as background set.

-TF_FC = Transcription factor fold change in the DESeq2 analysis

-TF_HigherIn = Muscle with highest expression based on fold change

-TF_DE_VLvsTA = Whether the gene’s DESeq2 adjusted p-value for differential expression between VL and TA is < 0.05. #N/A indicates those genes not included in the DESeq2 statistical analysis.

-Genes = Differentially expressed genes in the transcription factor category

-Columns M through R: The genes in column L are further listed based on the 3 main discussed categories and the muscle with highest expression.

**Supplementary Table S2E:** EnrichR significant signaling pathways from KEGG_2021_Human for all genes higher in VL and the 22414 expressed genes as background set.

**Supplementary Table S2F:** Significant isoform switches from IsoformSwitchAnalyzeR. A complete description of the column values can be found in: https://rdrr.io/bioc/IsoformSwitchAnalyzeR/man/switchAnalyzeRlist.html

-condition_1 = VL

-condition_2 = TA

-dIF = difference in isoform fraction (IF) = IF_2-IF_1

-abs_dIF = absolute difference in isoform fraction

-HigherUsage = muscle with higher usage

-isoform_switch_q_value = q-value of the test for differential isoform usage between the condition_1 (VL) and condition_2 (TA)

-gene_switch_q_value = q-value of the test for differential isoform usage in the gene between condition_1 (VL) and condition_2 (TA)

**Supplementary Table S2G:** EnrichR significant GO terms for Biological Process (GO_BP) and Cellular Component (GO_CC) for all 868 differentially expressed genes and the 17183 expressed genes as background set. GO terms overlapping with those enriched in the VL vs TA analysis are indicated in column K (Overlap_VLvsTA).

**Supplementary Table S3.** DESeq2 differential gene expression analysis of DMD versus Healthy.

-Gene type = Ensembl automatic biotype annotation for genes

-Transcription factor family = For transcription factor genes in AnimalTFDB v3.0, the family is indicated. NA = not in the database.

-Median TPM = median TPM across all TA samples

-Median TPM DMD = median TPM among the DMD TA samples

-Median TPM Healthy = median TPM among the Healthy TA samples

-baseMean = average counts

-pvalue adjusted = DESeq2 adjusted p-value. NA = not included in the DESeq2 analysis.

-Overlap VL vs TA = Genes that are significantly differentially expressed in both VL versus TA and DMD versus Healthy analyses

-Direction = Genes that are significantly upregulated in DMD and higher in VL (UpDMD_VL), or downregulated in DMD and higher in TA (DownDMD_TA)
